# Functional Characterization of a Novel Outer Membrane Porin *KpnO*, Regulated by PhoBR Two-Component System in *Klebsiella pneumoniae* NTUH-K2044

**DOI:** 10.1371/journal.pone.0041505

**Published:** 2012-07-25

**Authors:** Vijaya Bharathi Srinivasan, Manjunath Venkataramaiah, Amitabha Mondal, Vasanth Vaidyanathan, Tanvi Govil, Govindan Rajamohan

**Affiliations:** Council of Scientific Industrial Research, Institute of Microbial Technology, Sector 39 A, Chandigarh, India; University of Illinois at Chicago College of Medicine, United States of America

## Abstract

**Background:**

The diffusion of antibiotics through the outer membrane is primarily affected by the porin super family, changes contribute to antibiotic resistance. Recently we demonstrated that the CpxAR two-component signaling system alters the expression of an uncharacterized porin OmpC^KP^, to mediate antimicrobial resistance in *K. pneumoniae*.

**Principal Findings:**

In this study, functional characterization of the putative porin OmpC^KP^ (denoted *kpnO*) with respect to antimicrobial susceptibility and virulence was evaluated by generating an isogenic mutant, Δ*kpnO* in a clinical isolate of *K. pneumoniae*. Estimation of uronic acid content confirmed that Δ*kpnO* produced ∼2.0 fold lesser capsular polysaccharide than the wild-type. The Δ*kpnO* displayed higher sensitivity to hyper osmotic and bile conditions. Disruption of *kpnO* increased the susceptibility of *K. pneumoniae* to oxidative and nitrostative stress by ∼1.6 fold and >7 fold respectively. The loss of the *Klebsiella* porin led to an increase in the minimum inhibitory concentration of tetracycline (3-fold), nalidixic acid (4-fold), tobramycin (4-fold), streptomycin (10-fold), and spectinomycin (10-fold), which could be restored following complementation. The single deletion of *kpnO* reduced the survival of the pathogen by 50% when exposed to disinfectants. In *Caenorhabditis elegans* model, the *kpnO* mutant exhibited significantly (P<0.01) lower virulence. To dissect the role of PhoBR signaling system in regulating the expression of the *kpnO*, a *phoB* ^KP^ isogenic mutant was constructed. The *phoB* ^KP^ mutant exhibited impaired gastrointestinal stress response and decreased antimicrobial susceptibility. The mRNA levels of *kpnO* were found to be 4-fold less in *phoB* ^KP^ mutant compared to wild type. A regulatory role of PhoB^KP^ for the expression of *kpnO* was further supported by the specific binding of PhoB^KP^ to the putative promoter of *kpnO*.

**Conclusions and Significance:**

Loss of PhoBR regulated porin KpnO resulted in increased antimicrobial resistance, increased susceptibility to gastrointestinal stress, and reduced virulence in *K. pneumoniae* NTUH-K2044.

## Introduction

The cell envelope of Gram-negative bacteria consists of three principal layers: the outer membrane (OM), the peptidoglycan cell wall, and the inner membrane [1]. The OM is punctuated by a family of proteins, called outer membrane proteins (OMP) or porins [2], [3]. In *E. coli* and related gamma-proteobacteria, the major OMPs are OmpF (∼35 kDa), OmpC (∼36 kDa) and PhoE, and they differ in their solute selectivity, porin activity and gene expression in response to many environmental factors, such as osmotic pressure, temperature and pH [4]. The OM of Gram-negative bacteria plays a significant role in a variety of functions; it serves as a diffusion barrier to extracellular solutes and interacts with the bacterial environment. Influx is largely controlled by porins that are represented in large amounts in the OM. They form water-filled open channels that span the OM and allow the passive penetration of small hydrophilic molecules (>600 Da), such as iron, nutrients, and clinically significant antibiotics, such as β-lactams, aminoglycosides, carbapenems and fluoroquinolones [5]. Porins also serve as receptors for bacteriophages and bacteriocins and, in conjunction with peptidoglycan and LPS, play a significant role in maintaining the integrity of bacterial cells. As the major components of the OM, pore-forming proteins play a role in bacterial pathogenesis, such as adherence, invasion, and serum resistance [6].

Alterations in OM permeability, including modification of porin expression, have emerged as the major multidrug resistant (MDR) mechanism in key Gram negative clinical pathogens, such as *Escherichia coli*, *Salmonella* spp., *Enterobacter* spp., *Campylobacter* spp., *Acinetobacter baumannii*, and *Pseudomonas* spp., including the notoriously extreme drug resistant *Klebsiella pneumoniae* [7], [8]. *K. pneumoniae* are opportunistic pathogens and can give rise to severe diseases such as septicemia, pneumonia, urinary tract infections, and soft tissue infections [9]. The hospitalized, immunocompromised patient with underlying diseases is the main target of these bacteria. Thus, *Klebsiella* infections may serve as a paradigm of hospital-acquired infections [10]. Their incidence of 5 to 7% of all hospital-acquired infections ranks them among the most important nosocomial pathogen. *Klebsiella* is well known to most clinicians as a cause of community-acquired bacterial pneumonia occurring particularly in chronic alcoholics and showing characteristic radiographic abnormalities [11]. Carbapenems and cephalosporins are the most common drugs of treatment for *K. pneumoniae* infection however prevalence of MDR strains have led to failures of drug therapy [12].

**Figure 1:**
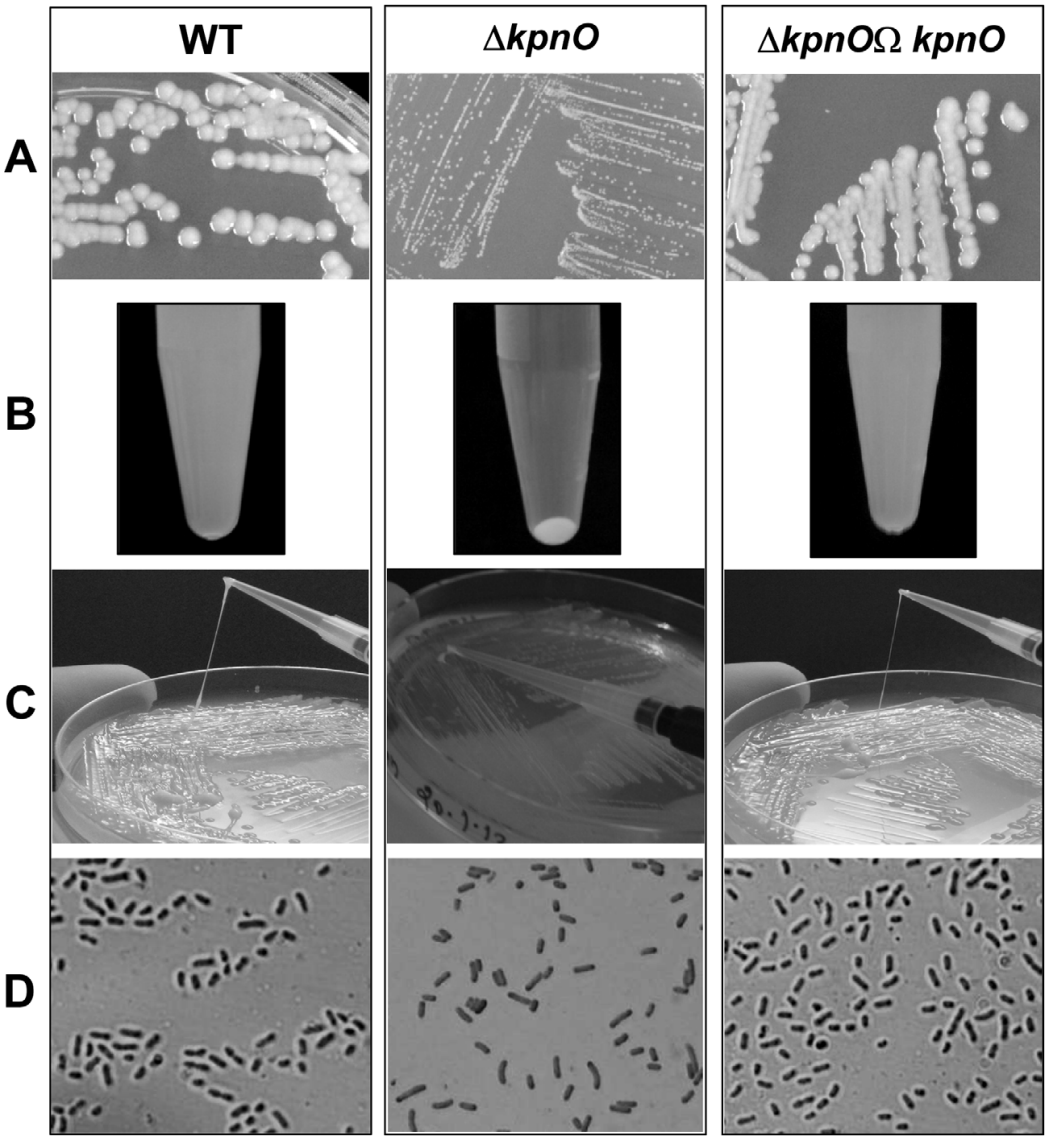
Phenotypic characterization of *kpnO* mutant. A) The *kpnO* mutant (Δ*kpnO*) had different colony morphology with smaller (0.75±0.05 mm on average) and less regular colonies than the wild-type (WT) (3.0±0.5 mm on average). B) The hypermucoviscosity string test was performed by measuring the formation of a viscous string stretched between the bacterial colony and the inoculation loop. The average lengths of WT, Δ*kpnO* were (5.0±0.5 cm) and (0.51±0.02 cm) respectively. C) The precipitation test was carried out on 12 h grown strains in LB broth at 37°C, and each pellet was evaluated after centrifugation at 4000rpm for 3 min. The WT and Δ*kpnO* showed profound difference in the compactness of the pellet. D) Cells were grown to late log phase in shaking culture and stained with crystal violet followed by treatment with 20% copper sulphate solution. The WT and Δ*kpnO* were visualized in Olympus microscope work station.

Similar to many enterobacterial pathogens, OmpK36 (OmpC homolog) and OmpK35 (OmpF homolog) are the two non-specific porins produced by *K. pneumoniae* through which nutrients and other hydrophilic molecules, such as carbapenems and cephalosporins, diffuse into the bacteria. Epidemiological studies indicate that porin loss in *K. pneumoniae* strains that produce ESBLs (Extended spectrum β-lactamases) display resistance to cefoxitin, oxyimino cephalosporins, monobactams, and fluoroquinolones [13]. A few studies have shown that strains producing CTX-M or AmpC type β-lactamases together with porin loss exhibit decreased susceptibility to carbapenems [14].

**Table 1:** Determination of capsular polysaccharides.

Strain	Glucouronic acid content (µg/10^9^ CFU)^a^	Mucoviscosity^b^
WT	18.14±1.09	+++
Δ*kpnO*	7.31±1.34	–
Δ*kpnO* Ω*kpnO*	15.07±1.26	+++
Δ*phoB* ^KP^	9.86±2.01	–
Δ*phoB* ^KP^ Ω*phoB* ^KP^	17.24±0.98	+++

a Values are the averages of triplicate samples represented by mean ± standard deviation.

b Confirmed by string test.

*K. pneumoniae* might express additional porins, such as PhoE, LamB, and OmpK37, which may be pivotal for normal cellular function in the absence of major porins OmpK36/35 [15]. In a previous study it was shown that absence of major porins OmpK35/K36 allows *K. pneumoniae* CSUB10S clinical isolate to exhibit high levels of resistance to various classes of antibiotics such as cefepime (8-fold), and cefotaxime (8-fold) and LamB deficiency in such OmpK35/K36 deleted background further increased the MICs of cefepime (16-fold) and cefotaxime (16-fold) respectively [16]. In another study, increased carbapenem resistance in OmpK36/K35 deficient clinical isolates has been correlated with down regulation of *phoE* in *K. pneumoniae* [17]. The alternative porin OmpK37 expressed at very low levels under standard laboratory conditions is known to have a minimal role in antimicrobial resistance [18]. Garcia-Sureda *et al* has recently reported that expression of the oligogalacturonate-specific porin OmpK26 compensated for the absence of OmpK35/36 in carbapenem resistant *K. pneumoniae* [19].

The *K. pneumoniae* NTUH-K2044 is hyper virulent clinical isolate with a thick capsule and has been isolated from a Taiwanese liver abscess patient, with *magA* and *rmpA* genes in its genome [20]. All these factors make this strain very suitable as a model organism for genomic studies. As for most bacterial pathogens, *K. pneumoniae* virulence is multifactorial, and there are many virulence factors that contribute to different disease syndromes. The success of this important pathogenic serotype in the varied ecological niches it can occupy depends on its ability to respond to the environment by differential regulation of its many virulence factors. Therefore understanding the biology of this human pathogen from the highly virulent serotype is critical to combat *K. pneumoniae* illness.

Previously we demonstrated that CpxAR two-component signaling system (TCS) alters the expression of a hypothetical porin (OmpK36 homolog) to mediate antimicrobial resistance in *K. pneumoniae* [21]. The current study was initiated to unravel the functions of the uncharacterized porin OmpC^KP^ (denoted *kpnO*) with respect to bacterial physiology in general and antimicrobial susceptibility in particular in *K. pneumoniae* NTUH-K2044 for the first time. *In silico* analysis revealed PhoB binding sites in the *kpnO* regulatory region, therefore, a *phoB* null mutant was constructed to evaluate regulation of the porin by the PhoBR TCS in *K. pneumoniae*.

**Figure 2:**
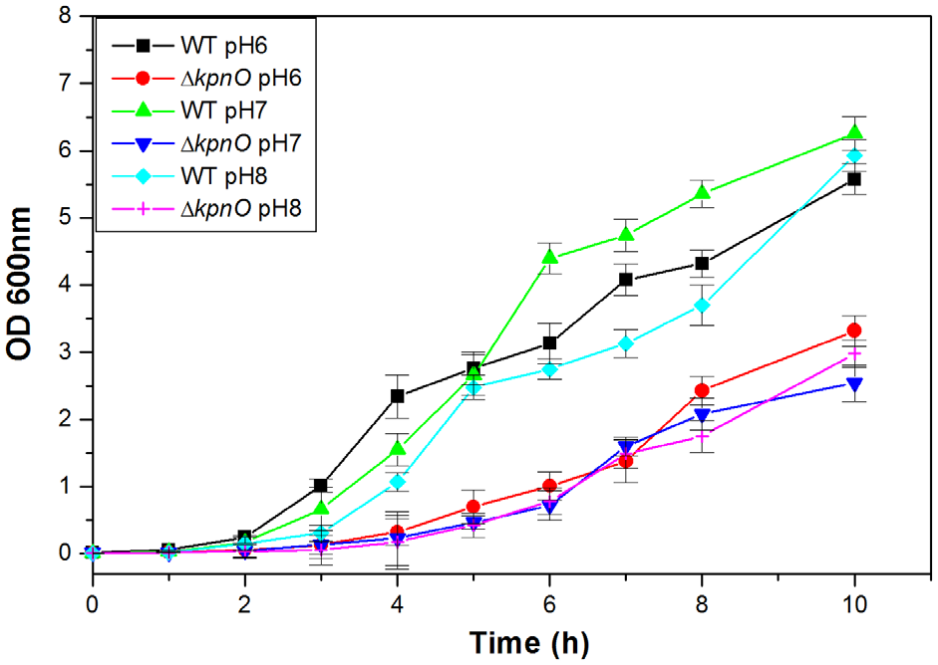
Growth kinetics. Growth kinetics of WT, and Δ*kpnO* was assessed in LB medium pH 6.0, 7.0 and pH 8.0.

**Figure 3:**
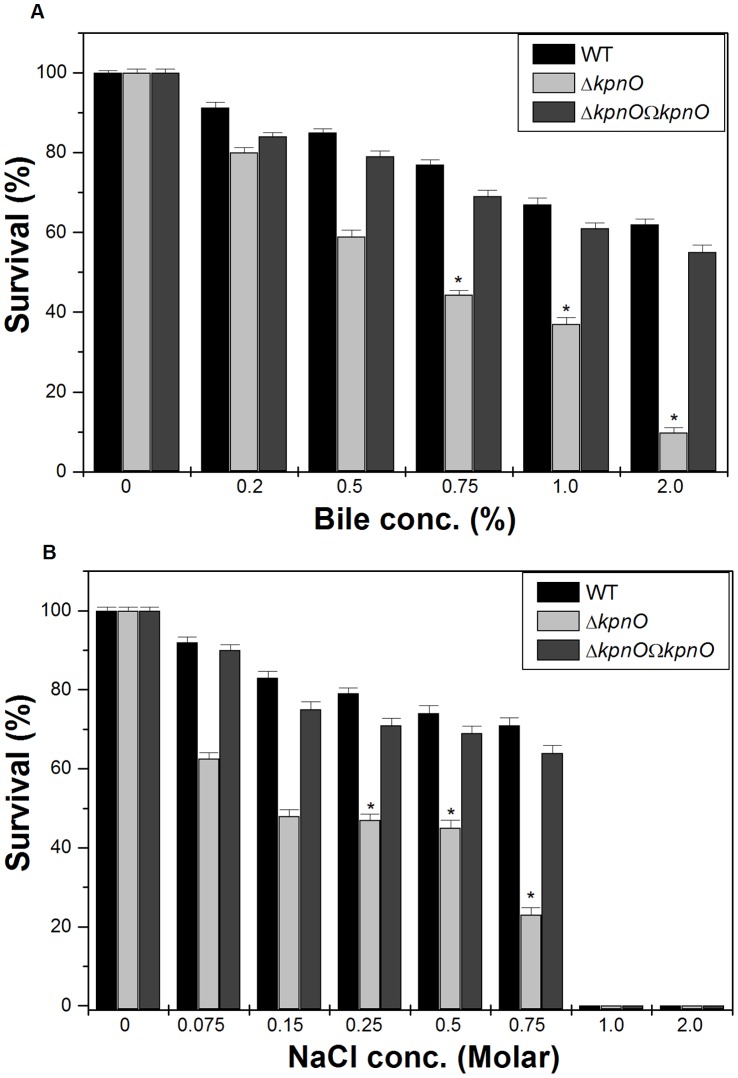
Bile and Osmotic challenge assays. A) Stress sensitivity of the WT wild-type strain, the *kpnO* mutant. The percentage of resistance to bile (0.2%, 0.5%, 0.75%, 1.0%, and 2.0%) was calculated by comparison to the numbers of viable cells in LB medium alone. B) The percentage of resistance to different concentration of NaCl stress (0.075 M, 0.15 M, 0.25 M, 0.5 M, 0.75 M, 1.0 M and 2.0 M) for WT and Δ*kpnO* was calculated by comparison to the numbers of viable cells in control.

## Results

### Bioinformatic Analysis of KpnO

The nucleotide sequence deduced from 1098 bp DNA fragment obtained from *K. pneumoniae* NTUH-K2044 shared >80% identity with the OmpC porins from other Gram negative pathogens [20]. The protein sequence of KpnO exhibits the following identities (identities in brackets) with OmpC from other *Klebsiella* strains such as *K. pneumoniae* 342 (Accession no: NC_011283.1) (96%), *K. pneumoniae* MGH78578 (Accession no: NC_009648) (95%), *K. pneumoniae* HS11286 (95%) and other bacteria such as *Enterobacter aerogenes* (89%), *E. coli* (83%), *Citrobacter youngae* (83%), and *Shigella dysenteriae* (81%) respectively. Regulatory elements that have been defined for *E. coli ompC*, could be found upstream to the start codon of *kpnO* including Fnr (230–243 bp), OxyR (262–307 bp), OmpR (358–367 bp) binding sites, as analysed by virtual footprint promoter analysis www.prodoric.tu-bs.de.

**Figure 4:**
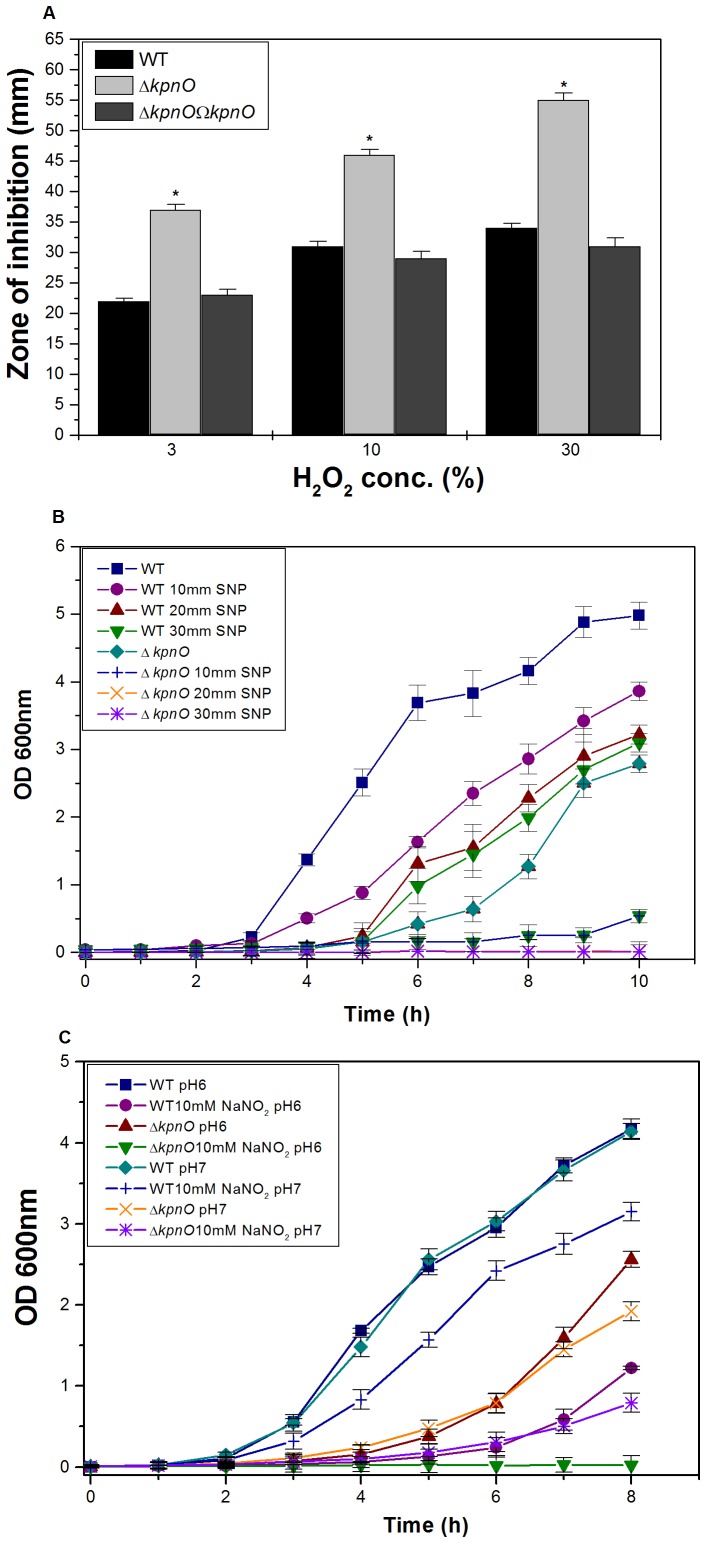
Oxidative and nitrostative challenge assays. A) Oxidative stress response of *kpnO* mutant. The ability of WT, Δ*kpnO* to combat different levels of hydrogen peroxide stress (3%, 10% and 30%) was measured by disc diffusion assay. The *kpnO* mutant displayed greater sensitivity to 30% H_2_O_2_ (inhibition zone  = 55±2.0 mm) than the wild-type (inhibition zone  = 34±0.0 mm). The data is the means of measurements made in triplicate and performed three times. *, Significant difference (P<0.05, Student t test). B) Effect of SNP at 10 mM, 20 mM and 30 mM concentration on growth of WT and Δ*kpnO*. Growth kinetics of Δ*kpnO* cells were ∼7 fold lower than the WT in the presence of 10 mM SNP, Δ*kpnO* exhibited ∼100 fold stunted growth at 20 mM and 30 mM SNP. C) Growth pattern of WT, Δ*kpnO* in the presence of sodium nitrite. In the presence of 10 mM NO donor, growth kinetics of Δ*kpnO* cells was ∼4.0 fold and ∼64.0 fold lower as compared to WT at pH 7.0 and pH 6.0 respectively.

### Deletion of *kpnO* Decreases Capsular Polysaccharide Production

To determine the biological role of *kpnO*, a *kpnO* mutant was created by conjugation in the wild-type *K. pneumoniae* NTUH-K2044. We used insertion-duplication mutagenesis to interrupt *kpnO*, required for the synthesis of a functional porin. PCR followed by DNA sequencing was done to confirm the disruption of the gene in *K. pneumoniae*. RT-PCR analysis confirmed that mutations abolished the transcription of *kpnO* (data not shown). The *kpnO* mutant had a different colony morphology when compared to the wild type. The wild type (WT) strain produced bigger (3.0±0.5 mm) and heavily mucoid colonies while *kpnO* mutant (Δ*kpnO*) colonies were smaller (0.75±0.05 mm) and non-mucoid, indicating a direct decrease in capsular polysaccharides (CPS) production (Figure 1–A). To determine the role of *kpnO* in CPS production, the hypermucoviscosity string test was performed; this test assesses the formation of a viscous string. The length of the strings for WT and Δ*kpnO* were 5.0±0.5 cm and 0.51±0.02 cm respectively (Figure 1–B). The precipitation test was carried out on 12 h grown culture in LB broth at 37°C. The WT did not form a dense pellet after centrifugation at 4000rpm for 3 mins while the Δ*kpnO* formed compact pellet (Figure 1–C). Visualization of cultures using 20% CuSO_4_ as per Anthony's capsule staining methodology revealed distinct difference in the exopolysaccharide production around NTUH-K2044 and Δ*kpnO* (Figure 1–D), and complementation restored their morphology. Quantification of uronic acid content reconfirmed the same observation (Table 1). These data suggest that KpnO contributes significantly towards capsule production of *K. pneumoniae* NTUH-K2044.

**Figure 5:**
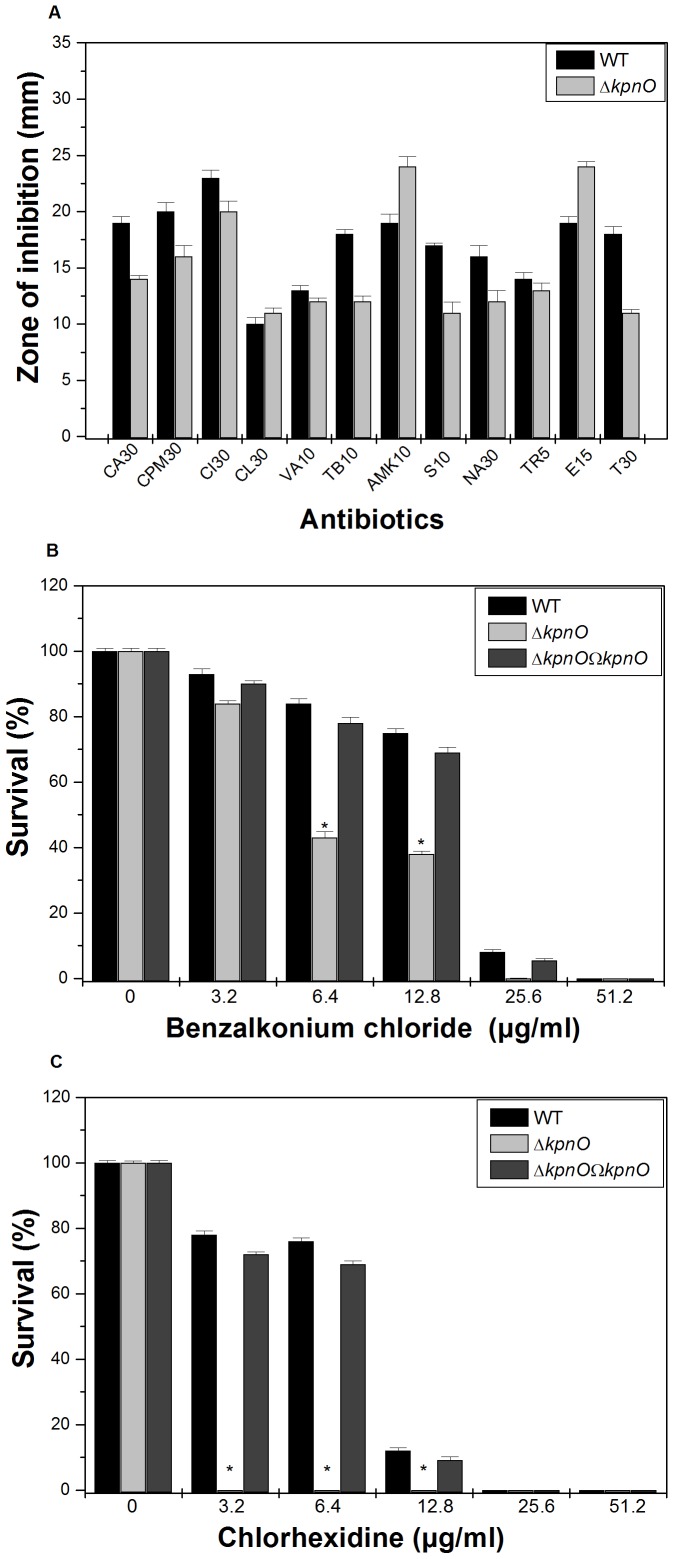
Antibiotic susceptibility testing and disinfectant challenge assays. A) The Kirby Bauer disc diffusion assay was performed with different antibiotics (CA30, CPM30, CI30, CL30, VA10, TB10, AMK10, S10, NA30, TR5, E15 and T30) using commercial discs. Data for representative drugs from each class have been shown here. B) Sensitivity towards benzalkonium chloride by WT and *phoB* ^KP^ mutant when cells were exposed to different concentrations of the disinfectant (3.2 µg/ml, 6.4 µg/ml, 12.8 µg/ml, 25.6 µg/ml, 51.2 µg/ml). C) Tolerance of WT and *phoB* ^KP^ to different concentration of chlorhexidine. The percent survival was calculated by comparison of viable cells in control. The datas are the means of measurements made in triplicate performed three times. *, significant difference (P<0.05, Student t test).

### KpnO Contributes to Growth and Gastrointestinal Stress Tolerance in *K. pneumoniae*

To decipher the involvement of KpnO in *K. pneumoniae* growth under conditions of different pH, the growth kinetics of Δ*kpnO* was compared with the wild type strain. Experimentally the growth characteristics of WT and Δ*kpnO* were determined over a period of ∼10 h in LB medium with different pH (3.0, 6.0, 7.0, 8.0 and 12.0) and subsequent data analysis revealed unique patterns. At pH 6.0, *kpnO* mutant exhibited reduced growth (>3.0 fold (±0.42) compared to the wild type after 6 h [WT/Δ*kpnO* mutant, P = 0.002]. The apparent density of Δ*kpnO* at pH 7.0 was 2.2 fold (±0.35) lower compared to wild-type after 10 h [WT/Δ*kpnO* mutant, P = 0.012]. At pH 8.0 Δ*kpnO* exhibited stunted growth (∼6 fold) compared to WT after 4 h and displayed a 2.0 fold density difference (Figure 2) [WT/Δ*kpnO* mutant, P = 0.004]. The other tested conditions of pH 3.0 and 12.0 were toxic to both the cultures. These results demonstrate that *kpnO* influences growth of *K. pneumoniae* under different pH conditions.

**Table 2:** Determination of MIC for WT, Δ*kpnO*, Δ*kpnO*Ω*kpnO*, Δ*phoB* ^KP^, and Δ*phoB* ^KP^Ω*phoB* ^KP^.

Antibiotics	WT	Δ*kpnO*	Fold change^a^	Δ*kpnO*Ω *kpnO*	Δ*phoB^KP^*	Fold change^a^	Δ *phoB^KP^*Ω *phoB^KP^*
Amikacin	0.064	0.032	2	0.064	0.008	8	0.064
Cefepime	2	4	2	2	0.5	4	2
Ceftazidime	0.256	0.512	2	0.256	0.128	2	0.256
Chloramphenicol	0.1	0.1	1	0.1	0.01	10	0.1
Colistin	0.01	0.01	1	0.01	0.001	10	0.01
Erythromycin	30	10	3	30	5	6	30
Nalidixic acid	0.1	0.4	4	0.1	0.1	1	0.1
Streptomycin	0.1	>1	10	>0.05	0.01	10	>0.05
Spectinomycin	0.1	>1	10	>0.05	0.1	1	0.1
Tetracycline	5	15	3	5	5	1	5
Tobramycin	0.1	0.4	4	0.1	0.1	1	0.1
Trimethoprim	0.1	0.1	1	0.1	0.01	10	0.1

E-strips were used to determine the precise MIC for different group of antibiotics such as ceftazidime, cefepime, ceftriaxone, nalidixic acid, streptomycin, tetracycline, trimethoprim, vancomycin, amikacin, erythromycin, tobramycin, chloramphenicol, ciprofloxacin, colistin, norfloxacin, ofloxacin, polymyxin, rifampicin, sparfloxacin, and spectinomycin following the CLSI guidelines. Complementation restored the MIC values. Units for MIC values are µg/ml.

a Fold change is the ratio of MICs for WT and Δ*kpnO* or Δ*phoB* ^KP^.

To determine the role of *kpnO* under conditions relevant to intestinal colonization, WT and Δ*kpnO* underwent specific gastrointestinal stress associated with bile and osmotic challenges. In the bile resistance assay, WT and Δ*kpnO* were exposed to different concentrations of bile (physiological concentration is 0.2% to 2%, [22]). The ability of WT to grow in the presence of 0.5% bile was 1.4 fold (±0.079), 0.75% bile was 1.7 fold (±0.017), 1% bile was 1.8 fold (±0.024) and 2% was 6.3 fold (±0.05) higher when compared to Δ*kpnO*, while transcomplemented Δ*kpnO*Ω*kpnO* strain restored the ability to tolerate bile stress (Figure 3–A) [WT/Δ*kpnO*, P = 0.018; WT/transcomplemented, P = 0.004]. The ability of WT to grow in the presence of NaCl (physiological concentration being 150 mM, [23]) at 0.25 M was 1.7 fold (±0.014), 0.5 M was 1.6 fold (±0.055), and 0.75 M was 3-fold (±0.44), higher when compared to Δ*kpnO* regardless of the inoculum size (Figure 3–B) [WT/Δ*kpnO* mutant, P = 0.014; WT/transcomplemented, P = 0.022]. To deduce the role of *kpnO* in temperature tolerance, we performed the heat shock assay. The temperature dependent assay showed that the *kpnO* mutant displayed 10% reduced survival compared to the wild type at 60°C (data not shown), thereby demonstrating the role of *kpnO* during temperature stress. [WT/Δ*kpnO* mutant, P = 0.42]. Overall results described in this section indicate that *kpnO* influences the response towards bile, osmotic and heat shock stress in *K. pneumoniae* NTUH-K2044.

**Figure 6:**
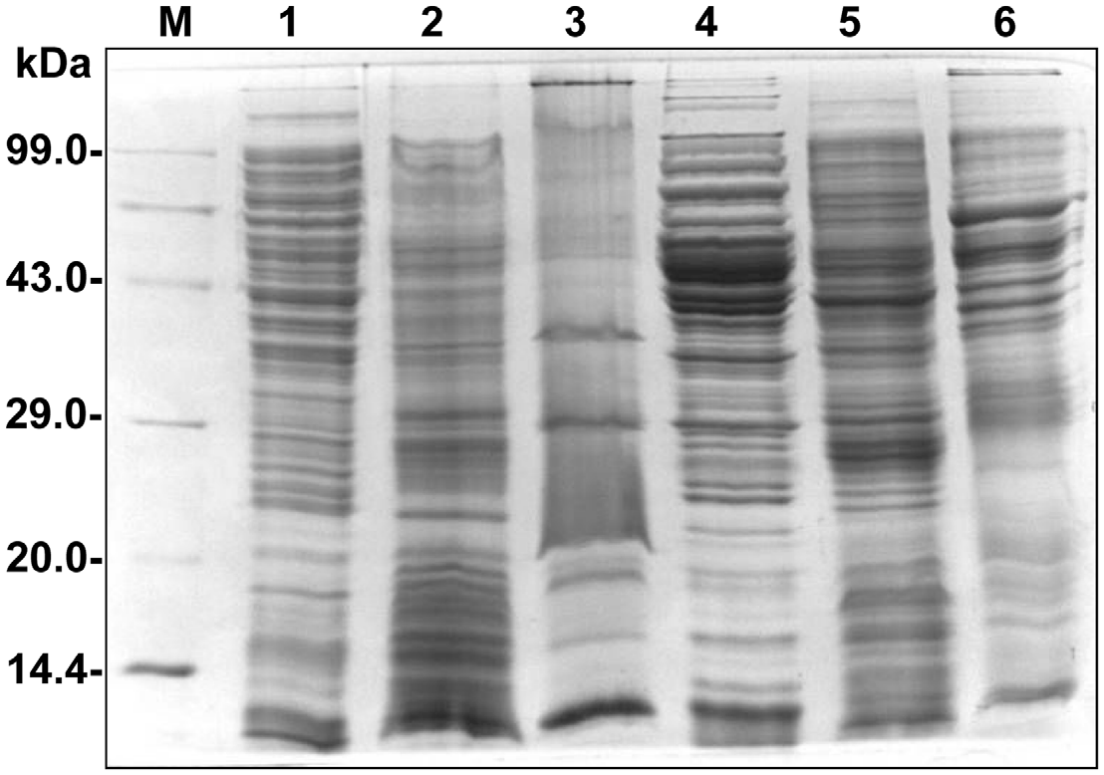
Protein profiling of WT and *kpnO* mutant strain. Membrane protein profiles were compared between the wild-type strain, and *kpnO* mutant. Total protein lysate of wild-type strain (lane 1), outer membrane fractions (lane 2), inner membrane fractions (lane 3), followed by total protein lysate of *kpnO* mutant (lane 4), outer membrane fractions (lane 5), inner membrane fractions (lane 6). Equal protein concentrations were separated by SDS-PAGE with a 5% stacking gel and a 12% separating gel and stained with coomassie brilliant blue. Lane M has molecular weight standards. The over expressed bands in outer membrane fractions of *kpnO* mutant are shown by arrow heads.

### Role of KpnO in Modulating Oxidative and Nitrostative Stress Response

To deduce the role of *kpnO* in oxidative stress, we performed the hydrogen peroxide challenge assays. Oxidative disc assay showed that *kpnO* mutant exhibited 1.6 fold greater sensitivity to 30% H_2_O_2_ (inhibition zone  = 55±2.0 mm) than the wild-type (inhibition zone  = 34±0.0 mm) (Figure 4–A) [WT/Δ*kpnO* mutant, P = 0.013; WT/transcomplemented, P = 0.38]. The sensitivity of stationary-phase cultures to oxidative stress was tested by exposing them to a range of H_2_O_2_ concentrations for 1 h. Only 47% of the Δ*kpnO* cells survived upon treating with 0.07894 mM hydrogen peroxide in comparison to the 95% survival observed in wild-type cells (Figure S1).

To test whether the presence of *K. pneumoniae* KpnO provides any protection against NO donor and nitrostative stress, we compared the growth profiles of WT and Δ*kpnO* in the presence of different concentrations of the NO donor sodium nitroprusside (SNP). Growth kinetics of Δ*kpnO* cells were ∼7 fold lower than the WT in the presence of 10 mM SNP [WT/Δ*kpnO* mutant, P = 0.01], growth was ∼100 fold lower at 20 mM [WT/Δ*kpnO* mutant, P = 0.02] and 30 mM SNP [WT/Δ*kpnO* mutant, P = 0.02] respectively (Figure 4–B). To further evaluate the function of *K. pneumoniae* KpnO in conferring susceptibility to other reactive nitrogen species, we tested tolerance of Δ*kpnO* towards acidified sodium nitrite. Protonated nitrite quickly degrades to generate numerous species of nitrogen oxides, for example nitric oxide [24]. The growth kinetics of Δ*kpnO* in the presence of 10 mM NaNO_2_ at pH 7.0 was ∼4.0 fold lower compared to WT [WT/Δ*kpnO* mutant, P = 0.017], whereas in the presence of acidified LB the growth was >64 fold lower in the mutant [WT/Δ*kpnO* mutant, P = 0.115] (Figure 4–C). The growth kinetics of Δ*kpnO* in the presence of 30 mM NaNO_2_ at pH 7.0 was ∼7 fold lower compared to WT [WT/Δ*kpnO* mutant, P = 0.048], while presence of 30 mM NaNO_2_ in acidified LB was toxic to both the strains [WT/Δ*kpnO* mutant, P = 0.026] (Figure S2). Results clearly indicate the role of *kpnO* in affecting oxidative and nitrostative stress response in *K. pneumoniae* NTUH-K2044.

**Figure 7:**
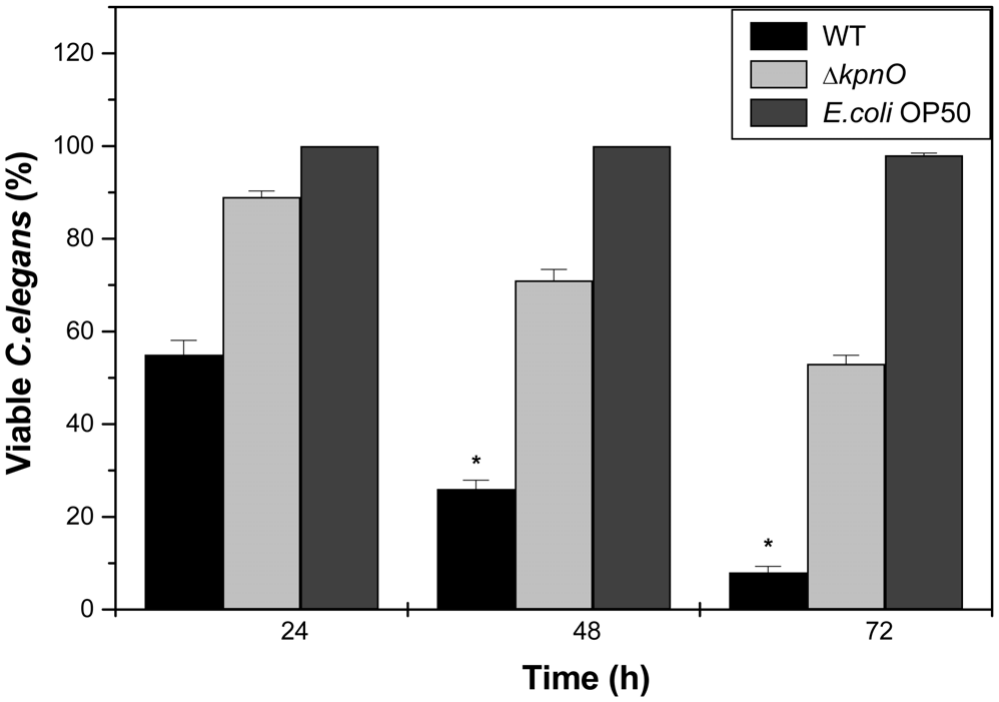
*C. elegans* killing assay. The *K. pneumoniae kpnO* gene is required for virulence to the nematode *C. elegans*. Survival of *C. elegans* after infection with *K. pneumoniae* WT, *kpnO* mutant and *E.coli* OP50.

**Figure 8:**
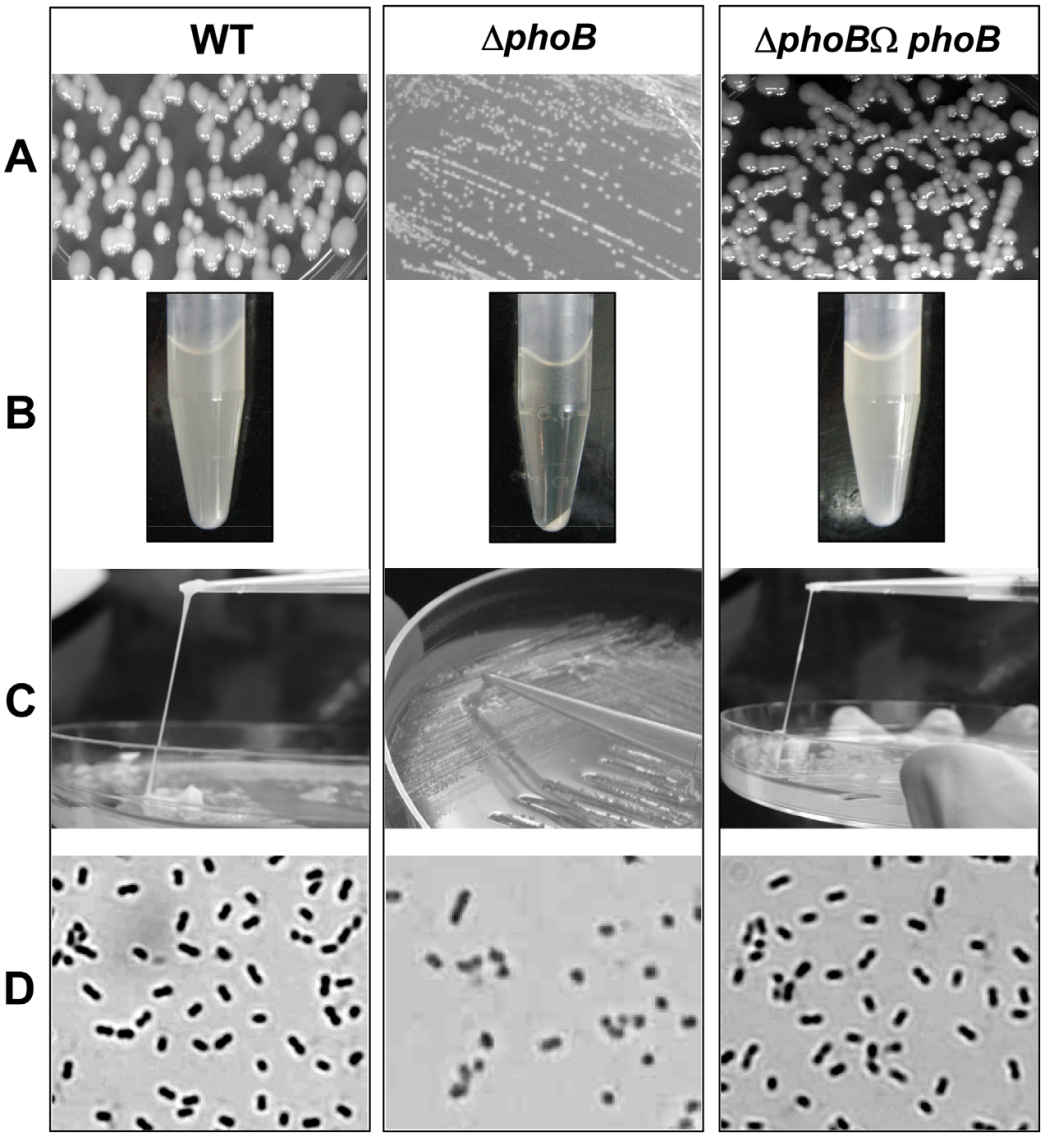
Phenotypic characterization of *phoB* ^KP^ mutant. A) Colony size of Δ*phoB* ^KP^ was smaller (1.0±0.15 mm) than the wild-type (3.2±0.7 mm). B) The *phoB* ^KP^ mutant produced smaller strings (0.75±0.02 cm) than wild-type (4.5±0.5 cm). C) Upon centrifugation at 4000 rpm for 3 min; Δ*phoB* ^KP^ formed dense pellet in contrast to WT. D) Δ*phoB* ^KP^ exhibited loss in capsular polysaccharide production as compared to WT.

### KpnO Mediates Antibiotic and Disinfectant Resistance by Altering Active Efflux

To evaluate the role of *kpnO* in drug resistance, antibiotic susceptibilities of WT and Δ*kpnO* were monitored. The results of disc diffusion assays indicated that upon deleting the porin, the bacterial cells displayed significantly altered susceptibility to ceftazidime, cefepime, ceftriaxone, tobramycin, amikacin, streptomycin, spectinomycin, nalidixic acid, erythromycin and tetracycline (Figure 5–A). The precise minimum inhibitory concentration (MIC) was further evaluated by following the guidelines of CLSI by E-test. The MIC of Δ*kpnO* was increased (fold increase in brackets); for different antibiotics namely nalidixic acid {4 fold}, tobramycin {4 fold}, streptomycin {10 fold}, spectinomycin {10 fold}, and tetracycline {3 fold} respectively compared to WT (Table 2). The MIC of erythromycin for Δ*kpnO* was 3-fold lower while compared to WT. In summary, deletion of *kpnO* altered the antibiotic susceptibility profile of *K. pneumoniae* belonging to K1 serotype.

To decipher whether *kpnO* confers antibiotic resistance by affecting drug efflux, screening for a potential efflux phenotype was accomplished by determining the growth profile of WT and Δ*kpnO* in the presence of antibiotics and carbonyl cyanide 3-chlorophenylhydrazone (CCCP) (10 µg/ml) as described in methods section. The growth rate of Δ*kpnO* in the presence of 0.005 µg/ml ciprofloxacin was >16 fold lower than the WT [WT/Δ*kpnO* mutant, P = 0.007]. The addition of CCCP drastically reduced the growth in both strains as the action of antimicrobials was restored (Figure S3). In independent experiments, growth remained unaltered on the addition of reserpine. Results described here demonstrated that deletion of *kpnO* impaired the active efflux capacity in *K. pneumoniae.*

**Figure 9:**
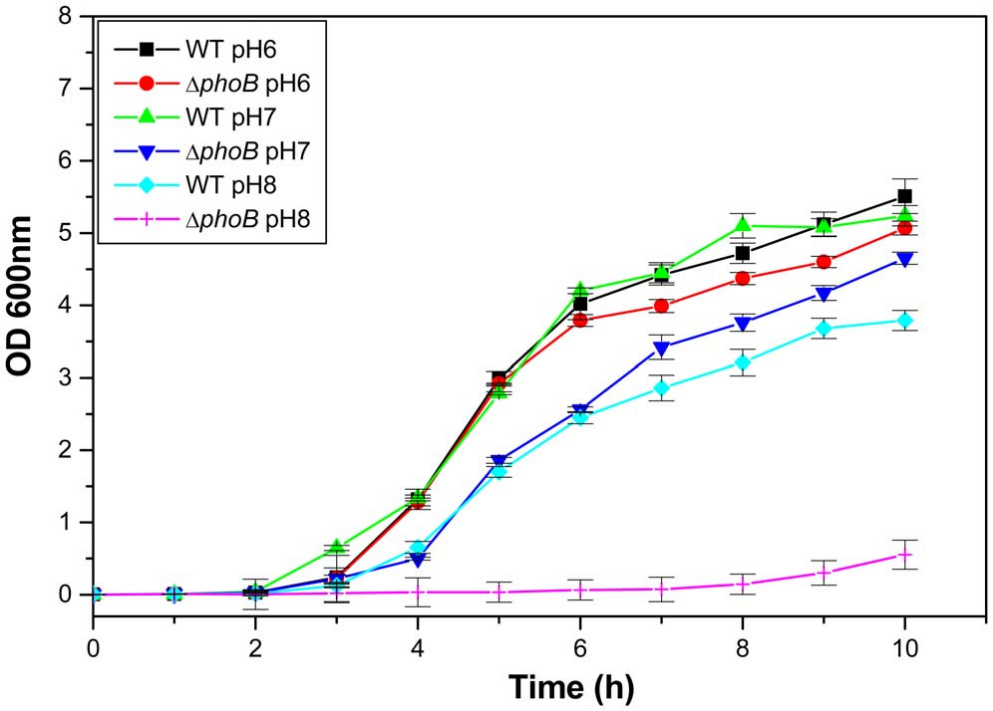
Growth kinetics. Growth kinetics of WT, and Δ*phoB* ^KP^ was assessed in LB medium pH 6.0, 7.0 and pH 8.0.

**Figure 10:**
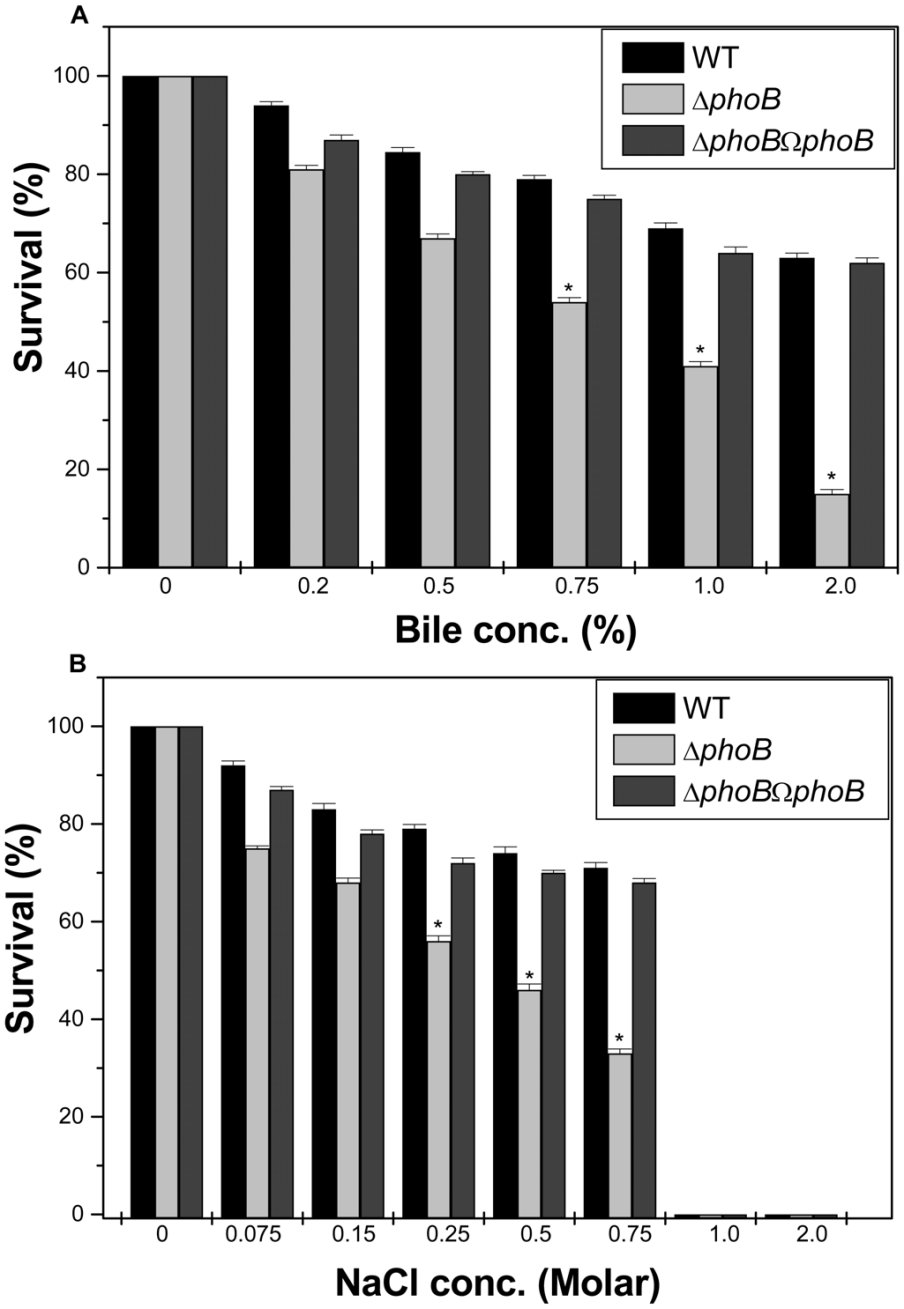
Bile and Osmotic challenge assays. Sensitivity of WT and *phoB* ^KP^ mutant to varied gastrointestinal challenges such as bile (A) and NaCl (B). The percent survival was calculated by comparison of viable cells in control.

*K. pneumoniae* is a nosocomial pathogen and has an ability to remain viable on abiotic surfaces for long periods of time [25]; therefore we tested the susceptibilities of WT and Δ*kpnO* towards different concentrations of popularly used hospital based disinfectants such as chlorhexidine and benzalkonium chloride. The percent survival of Δ*kpnO* cells was reduced by 50% when exposed to 6.4 µg/ml of benzalkonium chloride [WT/Δ*kpnO* mutant, P = 0.08; WT/transcomplemented, P = 0.04] (Figure 5–B). The percent survival of Δ*kpnO* was reduced by 100% when exposed to 3.2 µg/ml chlorhexidine [WT/Δ*kpnO* mutant, P = 0.13; WT/transcomplemented, P = 0.09] (Figure 5–C), indicating the contributory role of *kpnO* in conferring disinfectant resistance in this nosocomial pathogen. In conclusion involvement of *kpnO* in mediating antibiotic resistance *via* efflux mechanism and its added contribution towards disinfectant tolerance has been demonstrated for the first time in *K. pneumoniae*.

### Alterations in OM Profile of the *kpnO* Deletion Mutant in *K. pneumoniae*

The cell envelope is the prime target for most outside stress conditions that may modify envelope components and thus cause an extra cytoplasmic stress response [26], [27]. A reduction in the permeation of antibiotics is generally related to a decrease in porin expression or an alteration in the porin structure [28]. Thus, we compared the OMP profiles of Δ*kpnO* with WT to find out whether a *kpnO* deleted mutant expresses alternative porins/OMPs to maintain normal cellular functions. It was interesting to note that there was a marked difference in the OMP profiles of mutant compared to wild type (Figure 6), and currently our lab is involved in deciphering the identity and function of these proteins.

**Figure 11:**
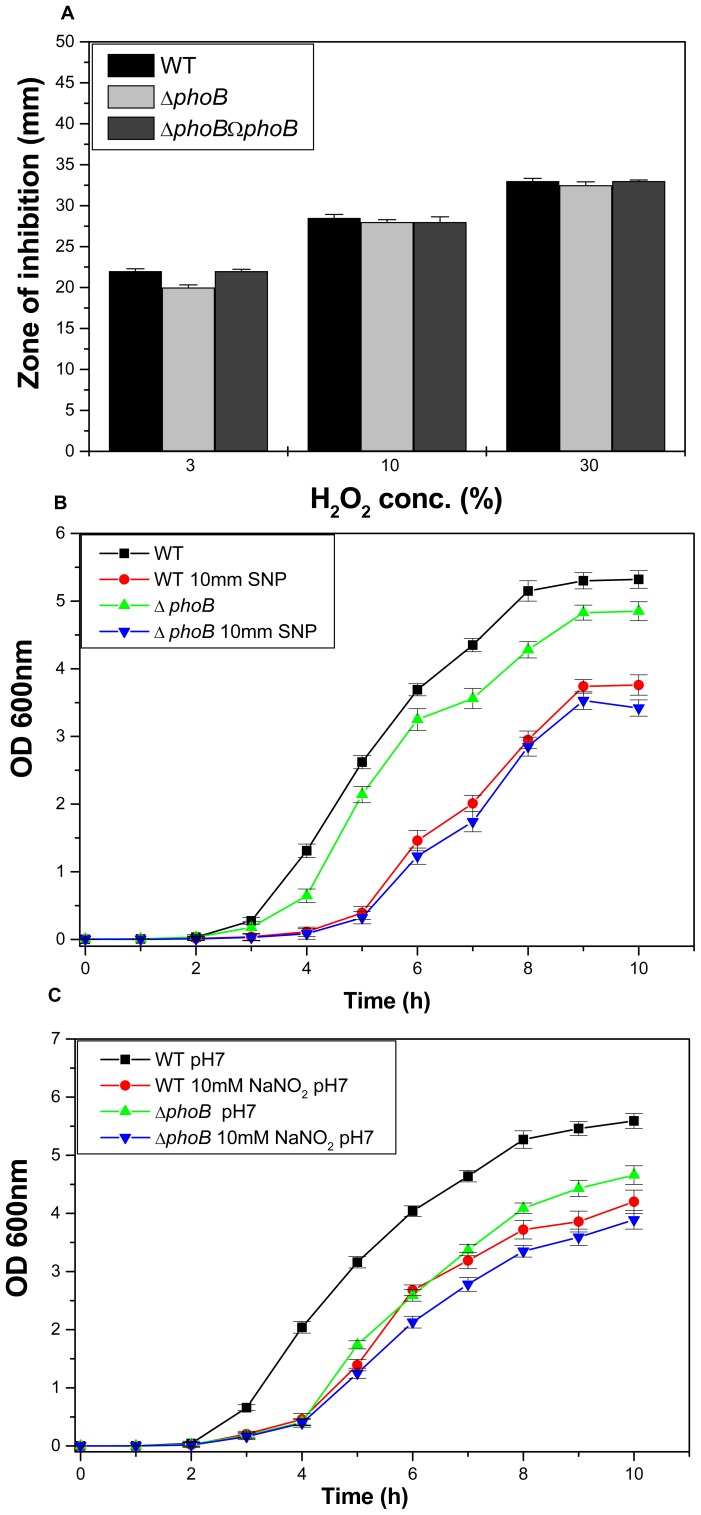
Oxidative and nitrostative challenge assays. A) Oxidative stress response of *phoB* ^KP^ mutant by disc assay. The *phoB* ^KP^ mutant displayed greater sensitivity to 30% H_2_O_2_ (inhibition zone  = 32.5±0.5 mm) than the wild-type (inhibition zone  = 33±1.0 mm). B) The data is the means of measurements made in triplicate. *, Significant difference (P<0.05, Student t test). B) Effect of SNP (10 mM) on growth kinetics of WT and Δ*phoB* ^KP^. C) Effect of NaNO_2_ (10 mM) on growth profile of WT and Δ*phoB* ^KP^.

### Role of KpnO in Virulence in *K. pneumoniae* NTUH-K2044

The *Caenorhabditis elegans* - *K. pneumoniae* infection model was employed to determine the involvement of *kpnO* in virulence. The wild type and mutant strains were examined for their abilities to kill *C. elegans.* The wild type strain displayed 80% and 90% killing at 48 and 72 h respectively. However, the mutant strain killed only 30% of the worms after 48 h (P<0.01) (Figure 7). Similar results were observed in liquid killing assay (data not shown). The *E.coli* strain OP50 was used as negative control. Thus, our findings demonstrate that the *kpnO* mutant kills *C. elegans* more slowly than wild type strain.

### Prediction of PhoB Binding Sites in Regulatory Regions of *kpnO*

Deciphering transcriptional regulatory systems is a key step in understanding the regulation of bacterial biological processes, as a whole. Genome sequence analysis of *K. pneumoniae* NTUH-K2044 revealed the presence of >466 signalling proteins (http://mistdb.com) including many uncharacterized TCS. Recently, we demonstrated that the CpxAR TCS that senses envelope stress alters the expression of *kpnO* to mediate antimicrobial resistance in *K. pneumoniae* [21]. Different stress response pathways are induced in bacteria under different environmental assails and one such important system is the PhoBR TCS, where PhoR is the histidine kinase (HK) and PhoB is the response regulator (RR) [29]. It is interesting to state here that on performing genome-wide prediction of *K. pneumoniae*, we identified putative PhoB binding sites in the *kpnO* regulatory regions. Given the association of the PhoBR regulatory system with stress responsive pathways and the apparent presence of a PhoB binding site upstream of *kpnO* caused us to investigate the probable role of PhoBR in regulating *kpnO*. Therefore, to evaluate the role of PhoBR system in antimicrobial susceptibility and stress response, a *phoB* ^KP^ mutant was constructed and expression of *kpnO* in the Δ*phoB* ^KP^ was monitored.

**Figure 12:**
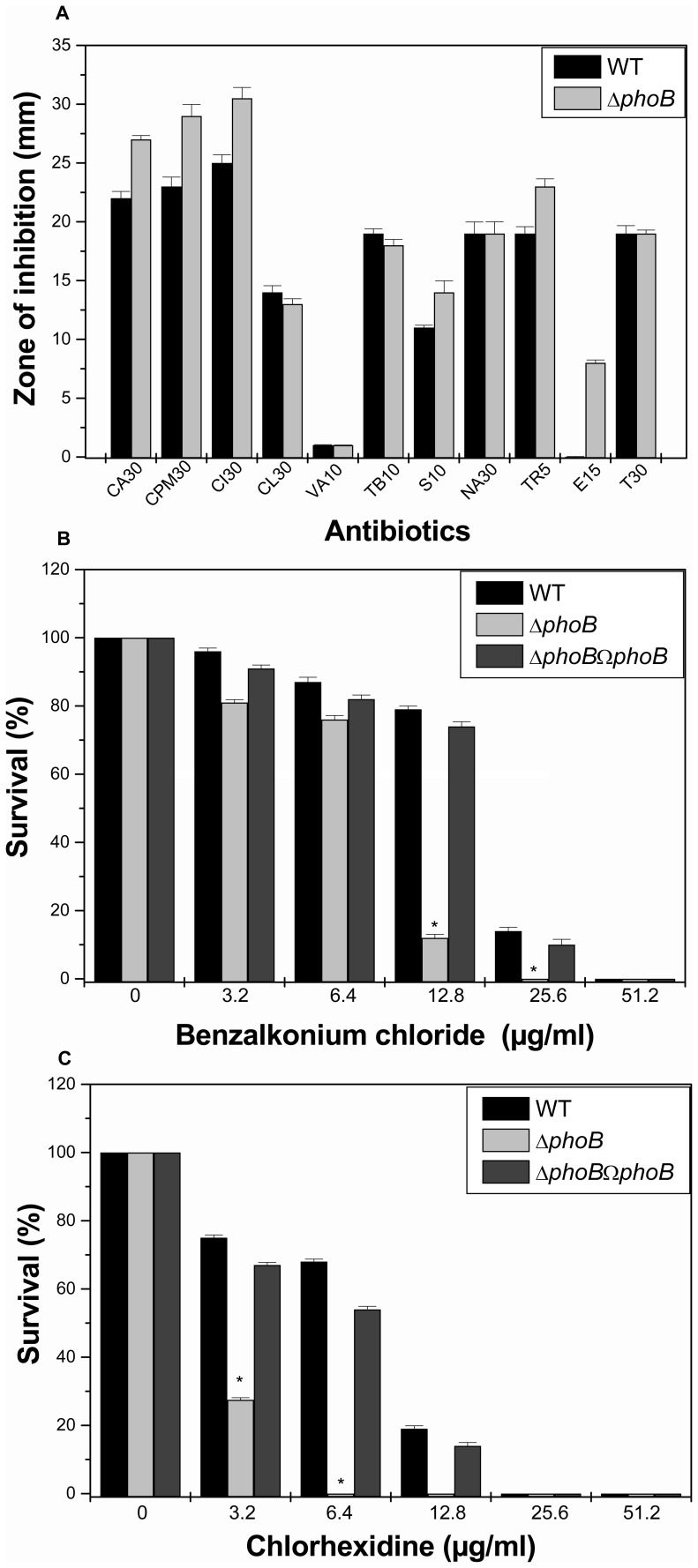
Antimicrobial susceptibilities of *phoB* ^KP^ mutant. Kirby Bauer disc diffusion assay was performed with different antibiotics and data for representative drugs are shown (A). Measure of disinfectant tolerance by WT and *phoB* ^KP^ mutant when cells were exposed to different concentrations benzalkonium chloride (B) and chlorhexidine (C). The percent survival was calculated by comparison of viable cells in control. The datas are the means of measurements made in triplicate performed three times. *, significant difference (P<0.05, Student t test).

**Figure 13:**
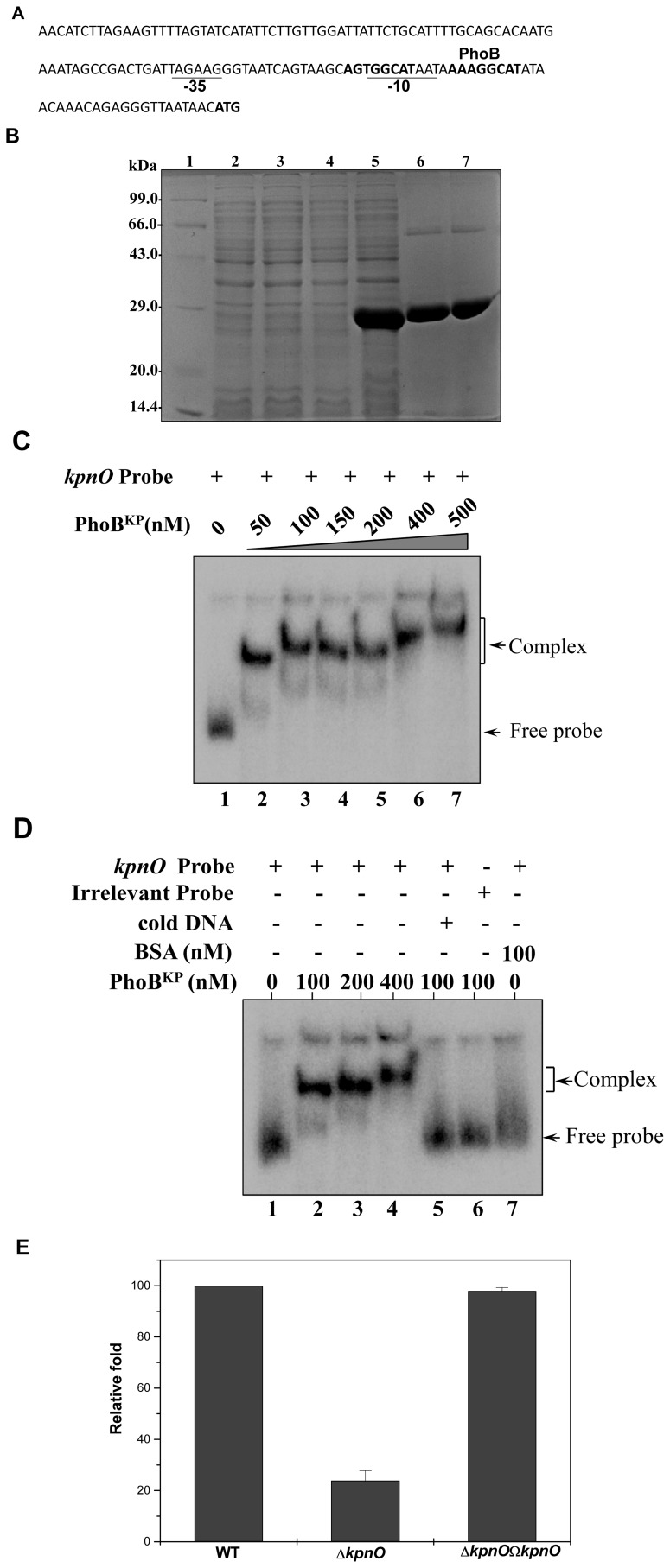
PhoB regulates *kpnO* in *K. pneumoniae*. A. Promoter region analysis of *kpnO*. The numbers in brackets represent the distance from the transcription start site. The −35 and −10 region in the promoter is underlined. Putative PhoB binding site has been shown in bold. B. SDS-PAGE profile of pET-*phoB* ^KP^. Lane 1: medium size marker, Lane 2: pET28C/BL21DE3 uninduced, Lane 3: pET28C/BL21DE3 induced, Lane 4: pET-*phoB* ^KP^/BL21DE3 uninduced, Lane 5: pET- *phoB* ^KP^/BL21DE3 induced, purified PhoB^KP^-fractions E1 and E2 (lanes 6–7) respectively. Protein samples after induction were subjected to SDS/PAGE (15% gel) followed by coomassie brilliant blue staining. C. Gel shift assays demonstrating the binding of PhoB to promoter of outer membrane protein *kpnO* in *K. pneumoniae* in a concentration dependent manner. Lane 1 (shows free probe), lanes 2–7 with increasing concentrations of PhoB protein (50 nM to 500 nM) respectively. Slower moving bound complexes and free probe has been indicated by arrows respectively. The gels are representative of at least three independent experiments. D. Gel shift assays demonstrating the sequence-specific binding of PhoB to *kpnO* using different controls as in lane 1 (shows free probe), lanes 2–4 (labeled *kpnO* promoter with increasing amount (100 nM, 200 nM and 400 nM) of PhoB), lane 5 (labeled *kpnO* promoter and PhoB with specific competitive inhibitor: 10 fold excess of unlabeled *kpnO* promoter), lane 6 (labeled non-specific DNA: promoter of *gyrA* and PhoB, 100 nM), lane 7 (labeled *kpnO* promoter with non-specific protein: BSA, 100 nM) respectively. The gels are representative of at least three independent experiments. E. Relative transcriptional level of *kpnO* in Δ*phoB* ^KP^ and Δ*phoB* ^KP^Ω*phoB* ^KP^ strains determined using real time RT-PCR is showed in comparison with wild type. The wild type expression level is represented as one fold. Each bar represents the average value of three independent experiments. Error bars are standard deviations.

### Deletion of *phoB* ^KP^ Affects Capsule Production and Cellular Growth

The colonies of WT were bigger (3.2±0.7 mm) with larger strings (4.5±0.5 cm) while the *phoB* ^KP^ mutant exhibited a reduction in colony size (1.0±0.15 mm) (Figure 8–A) with decreased mucosity by string test (0.75±0.02 cm) (Figure 8–B) and by centrifugation (Figure 8–C). Defects in exopolysaccharide production by Δ*phoB* ^KP^ were visually observed by Anthony's capsule staining (Figure 8–D) and reconfirmed by uronic acid quantification (Table 1). The *phoB* ^KP^ mutant exhibited stunted growth (∼6.8 fold) compared to WT in LB at pH 8.0 (Figure 9) [WT/Δ*phoB* ^KP^ mutant, P = 0.004]. The apparent density of Δ*phoB* ^KP^ was 1.6 fold (±0.35) lower compared to wild-type parent strain after 6 h, with no significant difference thereafter at pH 7.0 [WT/Δ*phoB* ^KP^ mutant, P = 0.001]. Results presented here demonstrate the role of *phoB* ^KP^ in capsule production and bacterial growth in *K. pneumoniae*.

### Impact of *phoB* Deletion on Gastrointestinal, Oxidative and Nitrostative Stress Tolerance

To determine the impact of the *phoB* deletion on general stress response, different stress challenge assays were performed. The ability of WT to grow in the presence of 0.75% bile was 1.5 fold (±0.027), 1% bile was 1.7 fold (±0.044) and 2% was 4.2 fold (±0.18) higher when compared to Δ*phoB* ^KP^, while the transcomplemented strain restored the ability to tolerate stress (Figure 10–A) [WT/Δ*phoB* ^KP^, P = 0.02; WT/transcomplemented, P = 0.02]. The ability of WT to grow in the presence of NaCl at 0.25 M was 1.4 fold (±0.033), 0.5 M was 1.6 fold (±0.076), and 0.75 M was 2 fold (±0.13), higher when compared to WTΔ*phoB* ^KP^ regardless of the inoculum size (Figure 10–B) [WT/Δ*phoB* ^KP^, P = 0.02; WT/transcomplemented, P = 0.017].

Oxidative stress tolerance remained unaltered in the *phoB* ^KP^ mutant of *K. pneumoniae* (inhibition zone  = 32.5±0.5 mm) as compared to wild type (inhibition zone  = 33±1.0 mm) [WT/Δ*phoB* ^KP^ mutant, P = 0.18] (Figure 11–A). The mutant remained unaffected when exposed to nitrostative stress conditions with 10 mM SNP [WT/Δ*phoB* ^KP^ mutant, P = 0.17] (Figure 11–B), 20 mM or 30 mM SNP respectively (Figure S4). The observation was the same upon using NaNO_2_ as the alternative NO donor (Figure 11–C). It is worthy to note that *phoB* ^KP^ deletion did not affect the capabilities of *K. pneumoniae* to tolerate oxidative and nitrostative stresses, but it displayed sensitivity to gastrointestinal like challenges.

### Loss of PhoB Diminishes Antibiotic and Disinfectant Susceptibilities

To evaluate the role of *phoB* in drug resistance, antibiotic susceptibilities of WT and Δ*phoB* ^KP^ were monitored. The results of disc diffusion assay displayed that upon deleting the TCS, the bacterial cells displayed altered susceptibility to ceftazidime, cefepime, ceftriaxone, ertapenem, carbenicillin, and the quinolones (Figure 12–A). The Δ*phoB* exhibited reduced MICs (fold decrease in brackets) to amikacin {8 fold}, cefepime {4 fold}, ceftazidime {2 fold}, chloramphenicol {10 fold}, Colistin {10 fold}, erythromycin {6 fold}, streptomycin {10 fold} and trimethoprim {10 fold} (Table 2) in comparison to WT. To monitor the impact on active efflux, the growth rate of Δ*phoB* ^KP^ in the presence of 0.005 µg/ml ciprofloxacin was monitored and data reflect a 1.2-fold lower growth in the mutant than the WT [WT/Δ*phoB* ^KP^ mutant, P = 0.001]. The addition of CCCP drastically reduced the growth in both strains as the action of antimicrobials got restored (Figure S5). The impact of PhoB deletion on biocide susceptibilities was assessed by survival assays. The WT cells survived up to 79% on exposure with 12.8 µg/ml of benzalkonium chloride while percent survival was only 12% with the *phoB* ^KP^ mutant [WT/Δ*phoB* ^KP^ mutant, P = 0.14; WT/transcomplemented, P = 0.02] (Figure 12–B). Like wise the WT cells survived up to 75% on exposure with 3.2 µg/ml of chlorhexidine while percent survival was only 27% in the *phoB* ^KP^ mutant [WT/Δ*phoB* ^KP^ mutant, P = 0.11; WT/transcomplemented, P = 0.11](Figure 12–C). Results described here demonstrated the involvement of *phoB* in mediating antibiotic and disinfectant resistance in *K. pneumoniae* for the first time.

### PhoB Regulates *kpnO* in *K. pneumoniae*

Inorganic phosphate is sensed and regulated in bacteria by the Pho regulon, which in turn is controlled by the PhoB-PhoR TCS [29]. PhoR, unlike other sensor HK, does not have a large periplasmic domain but has an extended cytoplasmic domain. The function of this extended cytoplasmic domain is proposed to sense internal signals that repress the kinase function of the PhoR. PhoB is the RR that binds to DNA and regulates transcription of genes, such as the high affinity phosphate-specific transport (pst) system, to acquire Pi [30]. Previous studies have reported that besides regulating the expression of the Pst system, Pho TCS also modulates the expression of other bacterial genes. PhoB binds to promoters that share an 18-bp Pho box of the sequence 5′-CTGTCATA(A/T)A(T/A) CTGT(C/A)A(C/T)-3′ [31].

We assessed the promoter region of *kpnO* and analysis revealed the presence of a conserved putative PhoB binding site spanning the region between 24 to 43 bp from the first methionine of KpnO (Figure 13–A). The PhoB^KP^; KP1_2137, is a 714 bp gene that encodes a polypeptide of 237aa (27 kDa). To define the possible interaction of PhoB^KP^ with the promoter of *kpnO*, we tested whether PhoB^KP^ directly interacts with the promoter region of *kpnO*; the *phoB* ^KP^ gene was cloned and expressed. The *phoB* ^KP^ gene from *K. pneumoniae* was PCR amplified, cloned into pET-28c and after transformation in *E. coli* strain BL21 (DE3), expression of the His-tagged protein (PhoB^KP^) was monitored following IPTG induction. Cell lysates were purified on a Ni-NTA column, and resolved by SDS/PAGE, subsequent analysis yielded an expected induced band of ∼27 kDa (Figure 13–B).

Thereafter, we carried out gel shift assays using the ^32^P-labeled *kpnO* promoter fragment. Protein-DNA complexes formed upon incubation of PhoB^KP^ with 500 bp radiolabelled *kpnO* promoter in reaction buffer, resolved on 5% PAGE revealed a clear retardation which was directly proportional to the protein concentration (Figure 13–C). Lack of any binding/retardation upon using different controls such as competitive (specific: 10 fold excess of cold promoter and non-specific: poly dI-dC) and non-competitive inhibitor (bovine serum albumin, BSA) clearly demonstrated the specific DNA binding ability of PhoB^KP^ to promoter region of *kpnO* in *K. pneumoniae* (Figure 13–D).

### Expression Analysis of *kpnO* in *phoB* ^KP^ Mutant in *K.pneumoniae*

Quantitative real-time RT-PCR (qRT-PCR) was used to examine expression of *kpnO* in wild-type, *phoB* ^KP^ mutant, and complemented strains. Compared to the wild-type strain, expression of *kpnO* was decreased by ∼4 fold in the *phoB* ^KP^ mutant (Δ*phoB* ^KP^ and wild type: *P*<0.0003, Student’s *t* test) (Figure 13–E). Complementation of the *phoB* ^KP^ mutation almost restored expression of *kpnO* (*P* values <0.0001). Together these results provide evidence for the regulatory role of PhoBR system on OMP KpnO.

## Discussion

OMPs allow for the passive diffusion of small molecules into the bacterial cell [32]. Passage of solutes through the cell envelope and control of this process are essential to cell survival when nutrients are scarce or when the cell is exposed to toxins or other adverse conditions [2]. In *E. coli*, there are three major OMPs, OmpA, OmpC, and OmpF, which function as passive diffusion channels for small molecules, such as nutrients, toxic salts, and antibiotics [26]. Changes in OMPs have been recognized to be very important in the development of clinical antibiotic resistance [28]. Mutations in OmpC have been dissected in *E. coli* and *Enterobacter aerogenes* that are isolated after drug treatment [33], [34].

In *K. pneumoniae* OmpK35 and OmpK36 are the two major porins [13]. Previous study has shown that combination of high-level production of AmpC β-lactamases such as ACT-1, CMY-4, DHA-1 or SHV-2 together with porin loss can result in resistance or reduced susceptibility to carbapenems in *K. pneumoniae* [35]–[37]. A separate study from Korea has shown that production of CMY-2 and DHA-1 together with loss of OmpK35 and OmpK36 resulted in an outbreak of *K. pneumoniae,* where strains remained mostly non-susceptible to imipenem [38]. In another study, the presence of CTX-M-1, together with loss of OmpK35 and OmpK36 was shown to result in resistance [39]. These findings suggest that OmpK36 plays an important role in resistance. The OmpK36 homolog is annotated in the genome sequence of hyper virulent and hyper mucoviscous strain, however its functions have remained completely unexplored so far. Thus experimental evidence for the biological functions of the OmpK36 homolog in *K. pneumoniae* NTUH-K2044 with respect to general bacterial stress response, antimicrobial resistance and virulence has been provided in this report for the first time.

The CPS of *K. pneumoniae* is complex acidic polysaccharide consisting of repeating units of 3–6 sugars. The type of sugars seems to correlate with the virulence, and untill now 78 capsule types have been identified [9]. The wild type strain was found to express a prominent capsule like structure surrounding the bacterial cell, while the *kpnO* mutant displayed a non-mucoid phenotype and lacked the well-defined capsule coat. In *K. pneumoniae* KpnO possibly functions as the auxiliary protein necessary for export of high molecular weight polysaccharides to the bacterial surface to form the capsule. Enteric bacteria are known to respond to hostile conditions in the host by altering the expression of genes whose products are involved in the resistance mechanism. The *kpnO* mutant displayed 1.5–3.0 fold higher sensitivity to varied gastrointestinal like stress irrespective of the inoculum size. Experimental evidence pinpointing the key role of KpnO in survival of the pleomorphic bacillus under conditions mimicking the upper parts of the GI, where they encounter hyper osmotic and bile salts condition in a microaerobic environment has been provided for the first time in *K. pneumoniae.* Impaired adaptation of *kpnO* during temperature stress reveals its key role in heat shock tolerance.

Antimicrobial therapy for *K. pneumoniae* is often ineffective as members of the *Klebsiella spp*. are highly resistant to most clinically relevant antimicrobial agents [12]. Multidrug resistance in *K. pneumoniae* is defined as resistance to all of the agents belonging to at least two of three classes of antibiotics, such as quinolones, aminoglycosides, and β-lactam agents [40]. A study in *S. Typhimurium* has shown that loss of porins STM1530 and OmpD were important for ceftriaxone resistance [41]. A recent study by Tsai *et al* in *K. pneumoniae* strain NVT2001 belonging K2 serotype has demonstrated that deletion of *ompK36* results in resistance to a group of β-lactams such as cefazolin, cephalothin, and cefoxitin [13]. Current study on the porin *kpnO* from the K1 serotype reflects its broad spectrum antibiogram. Loss of *kpnO* rendered cells resistant not only to β-lactams such as ceftazidime, cefepime, ceftriaxone, but also to aminoglycosides such as tobramycin, streptomycin, spectinomycin, including quinolone such as nalidixic acid and polyketide such as tetracycline.

In previous years, the possibility that widely-used disinfectants might co-select for antibiotic resistance has been suggested to pose a potential risk to the successful treatment of hospital acquired infections [42]. A few studies have shown that disinfectants induce resistance determinants thereby reducing susceptibility to antibiotics in bacteria [43]. Therefore, it is plausible that antibiotic sensitive *K. pneumoniae* commonly found in hospitals (where there is heavy use of disinfectants), when exposed to such continued selective pressure might transform to exhibit antibiotic resistant phenotypes. Besides, we found that *kpnO* mutant exhibited a reduced ability to kill the nematode *C. elegans* demonstrating its key role in virulence. Hence, these studies implicate an important role for *kpnO* in antimicrobial resistance and virulence.

Signal transduction systems are intracellular information processing pathways that translate external stimulus to an adaptive cellular response [44]. In our previous study, we showed that CpxR modulates the expression of *kpnO* to mediate antimicrobial resistance in *K. pneumoniae* [21]. The current study was expanded by decoding the regulation of *kpnO* in *K. pneumoniae* NTUH-K2044. In *E. coli* anaerobiosis is known to up regulate the expression of OmpC [32]. Previous reports have shown that acidic pH and presence of chemicals such as bile salts in the environment also induce expression of OmpC [45]. Another study has shown that OmpC is expressed under high osmolarity conditions in animal intestine at 37°C [46]. Thus one common signal transducing system that can sense all these varied signals is the PhoBR TCS in bacteria [30], [31]. Given that PhoBR TCS is a stress responsive signaling system and that the presence of PhoB binding site was detected upstream of *kpnO*, these observations suggested that PhoBR was a potential regulator of *kpnO.* Therefore, understanding the regulation of OmpC homolog by PhoB is quite intriguing as both the proteins are highly conserved in bacterial species. The constructed *phoB* ^KP^ mutant displayed impaired sensitivity to gastrointestinal stress similar to that of Δ*kpnO*. A significant reduction in *kpnO* mRNA level in *phoB* ^KP^ mutant, together with the binding of PhoB on the regulatory fragments of *kpnO* provide strong evidence for the involvement of PhoBR system in regulating the expression of *kpnO*. Interestingly, deletion of *kpnO* rendered the bacteria resistant to different classes of antibiotics, in contrast to the behaviour of Δ *phoB* ^KP^ where cells became sensitive. The well characterized TCS in bacteria EvgSA, BaeSR, CpxAR and PhoBR are capable of inducing efflux pumps which leads to decreased antibiotic susceptibility [8], [22]. The positive regulation of efflux genes *mdtABC* and *acrD* in *E. coli* and *S*. Typhimurium by TCS leading to increased resistance of β-lactams, novobiocin and deoxycholate has been reported previously [47]. With such documented observations, the possibility of altered/decreased efflux pump expression reducing antibiotic susceptibility/tolerance in Δ*phoB* ^KP^ cannot be ruled out. A study relating to the impact of *phoB* ^KP^ on efflux pump expression/activity in *K. pneumoniae* is highly warranted. To our knowledge, this is the first evidence showing that general bacterial porin *kpnO* regulated by PhoBR is involved in mediating resistance against GI stresses, affecting antibiotic/disinfectants susceptibilities in *K. pneumoniae* NTUH-K2044; hyper virulent K1 serotype.

## Materials and Methods

### Bacterial Strains, Plasmids and Media

*K. pneumoniae* NTUH-K2044 (This strain came from the blood of a previously healthy individual who was diagnosed with a community-acquired primary liver abscess and metastatic meningitis) was kindly provided by Dr. Jin Town Wang of the National Taiwan University Hospital, Taipei, Taiwan [20], [48]. *E. coli* S17-1λ pir which carries the F plasmid and encodes π protein essential for replication of pUT-Km was used for cloning experiments. Bacteria cultures were grown in Luria-Bertani (LB) broth or on LB agar (Difco, Becton-Dickinson, Sparks, MD) at 37°C with constant shaking (220 rpm) and supplemented with Kanamycin (100 µg/ml) where required.

### DNA Methods

Restriction digestion, ligation, transformation, and agarose gel electrophoresis were done according to standard protocols. Plasmids were prepared from *E. coli* using a QIAprep Spin miniprep kit from Qiagen according to the manufacturer's protocol. Mobilization of plasmids into *K. pneumoniae* cells was performed as previously described [21]. Genomic DNA of *K. pneumoniae* was extracted using the Gene Aid DNA purification kit according to the manufacturer's instructions. DNA fragments used for cloning were extracted from agarose gels using a QIA quick gel extraction kit from Qiagen. PCR products were purified using a QIA quick PCR purification kit (Qiagen) and, when cloned, sequenced to confirm the correct sequences (Applied Biosystems). Primers used in the present study were custom-synthesized (Eurofins MWG operons, Germany).

### Construction of Deletion Mutants in *K. pneumoniae* Strain NTUH-K2044

The MisT2 database (www.mistdb.com) shows the presence of 5262 proteins in the 5,472,672 bp (GC content: 57.4%) genome sequence of the K1 serotype (Accession No: AP006725.1). The putative OmpC homolog, KP1_3869 (denoted *kpnO*) is located starting from nucleotides 3698768 bp to 3699865 bps (*kpnO*: 1098 bp, 365aa) in the genome sequence of *K. pneumoniae* NTUH-K2044 [20]. To construct knock out, a 540 bp internal fragment was amplified by PCR using Δ*kpnO*-F and Δ*kpnO*-R primer from its genomic DNA (Table 3). The PCR product was ligated into an *EcoR*I digested plasmid pUT-Km which was blunted by klenow reaction that contained the kanamycin resistance gene, transformed into *E. coli* S17-1λ pir and the resulting recombinant plasmid harbouring the internal fragment of *kpnO* was designated as pUT-*kpnO*. The plasmid pUT-*kpnO* was mobilized into recipient *K. pneumoniae* NTUH-K2044 from donor *E. coli* S17-1λ pir.

**Table 3:** Primers used in this study.

Primer name	Primer sequences (5′-3′)
Δ*kpnO*-F	TTGGCGACGCGGGCTCTTTCGACTACGGTC
Δ*kpnO*-R	AAGCGCAGAACTTCGAAGTGGTTGCTCAGT
Primer NT-1	GAGTACATATGAAAGTTAAAGTACTGTCCCTCCTG
Primer CT-2	TACTAGGATCCTTAGAACTGGTAAACCAGGCC
*phoB*-F	GGTACATATGAGCGTCAGACTACTATCGAA
*phoB*-R	TCTAGGATCCTCAGCGCAGTTCGAACAGAT
prom *kpnO*-F	GGCCTAATTGATTGATTAATAGTCGTTAGGGAAT
prom *kpnO*-R	GTTATTAACCCTCTGTTTGTTATATGCCTTTTAT
Δ*phoB^KP-^*F	GGAAGCGGACTACTATCTGGGCGAACATCTCC
Δ*phoB^KP^*-R	TCAGGGTTTCCATAATGGTGTATTCAAAGGCG
Primer NT-3	GAGTACATAATCACCAACCCGCTCGCCGTTCGCA
Primer CT-4	TACTAGGATCCATGCCGTAGGCCAGGGAGAGCA
RT-*kpnO* ^KP^NT	ACCCAGACCTACAACGCAAC
RT-*kpnO* ^KP^CT	ATTTCAGGATGTCCTGGTCG
RT-*rpoB* ^KP^NT	GCGGTTGGTCGTATGAAGTT
RT-*rpoB* ^KP^CT	TGGCGTTGATCATATCCTGA

Briefly, *K. pneumoniae* was inoculated into 10 ml LB and was incubated for 2–3 h till OD_600 nm_ reaches 0.2. For matings, recipient and donor culture were mixed in a ratio of 1∶2 respectively, pelleted and spotted onto the centre of an LB agar plate. After 3 h of growth at 37°C the cells were plated on *Klebsiella* selective agar (HiMedia HiCrome *Klebsiella* Selective Agar Base cat# M1573; *Klebsiella* Selective Supplement cat# FD225) containing Kanamycin 100 µg/ml and 5 µg/ml chlorhexidine to select for colonies. It is expected that colonies that appear on the selective plate would be transconjugants that resulted from one DNA exchange event in which the whole suicidal plasmid gets incorporated in the *K. pneumoniae* genome. The disruption at *kpnO* gene was confirmed with selected transconjugant by PCR and DNA sequencing using gene specific and genome flanking primers and deleted mutant was denoted as Δ*kpnO*. Intact *kpnO* gene was amplified along with its promoter using primer NT-1 and primer CT-2 and cloned into a pCRIITOPO-CAT plasmid (Table 3). The selected recombinant plasmid harbouring the intact *kpnO* gene was transformed into the *kpnO* isogenic mutant strain by electroporation. The complementation strains were selected on LB agar plates supplemented with 50 µg/mL kanamycin and 100 µg/mL chloramphenicol and transcomplemented strain was designated as Δ*kpnO*Ω*kpnO*.

The PhoB homolog, KP1_2137 (designated PhoB^KP^) is located starting from nucleotides 2081389 bp to 2082102 bp (*phoB^KP^*: 714 bp, 237aa) in the genome sequence of *K. pneumoniae* NTUH-K2044 [20]. A 380 bp internal fragment from *phoB^KP^* was amplified by PCR using Δ*phoB^KP^*-F and Δ*phoB^KP^*-R, and null mutant denoted Δ*phoB^KP^* was constructed following standard procedures as mentioned above. Intact *phoB^KP^* was amplified along with its native promoter using primer NT-3 and primer CT-4 and cloned into a pCRIITOPO-CAT plasmid. The selected recombinant plasmid harbouring the intact *phoB^KP^* gene was transformed into the respective isogenic mutant by electroporation to generate Δ*phoB^KP^*Ω *phoB^KP^*.

Mutant (Δ*kpnO* and Δ*phoB^KP^*) and complemented strains generated in this study (Δ*kpnO*Ω*kpnO* and Δ*phoB^KP^*Ω *phoB^KP^*) were characterized; their phenotypes were compared with the WT.

### Tests for Hypermucoviscosity

The mutant, complemented and WT strains were streaked onto LB agar plates and incubated at 37°C overnight. A standard bacteriologic loop was used to stretch a mucoviscous string from the colony. Hypermucoviscosity was defined by the formation of viscous strings >5 mm in length when a loop was used to stretch the colony on agar plate which was considered the positive string test [48]. The strains to be tested were cultured 12 h in LB broth at 37°C and subjected to centrifugation at 4000rpm for 3 mins to check reduction in mucoidy. For exopolysaccharide analysis [49], cells were grown to late log phase in shaking culture and stained with crystal violet followed by treatment with 20% copper sulphate solution (Anthony's capsule staining methodology). Samples were visualized using an Olympus microscope work station. CPS was extracted from 12 h grown bacterial suspensions adjusted to ∼10^8^ cells per ml with Zwittergent 3–14 detergent. The amount of uronic acid was then measured according to the method described previously [50]. Each experiment was performed in triplicate.

### *In vitro* Growth Curves

To examine bacterial growth *in vitro*, overnight cultures were diluted 1∶100 and subcultured for 10 h. The growth kinetics was monitored with LB at different pH (3.0, 6.0, 7.0, 8.0 and 12.0). The growth inhibition assay was performed as described previously [21]. The efflux pump inhibitors (10 µg/ml) used in this study was CCCP or reserpine (Sigma, St. Louis, MO). Efflux pump inhibitors had no intrinsic antibacterial activity against wild type strain at the concentration used in the experiments.

### Osmotic, Bile, Disinfectant Challenge Assays

Various stress assays were performed as described previously [50]. Briefly mutant, complemented and WT strains were grown to mid-exponential phase, cultures were spread onto LB agar plates containing different concentrations of NaCl (0.075 M, 0.15 M, 0.25 M, 0.5 M, 0.75 M, 1 and 2 M), bile (0.2%, 0.5%, 0.75%, 1.0%, and 2.0%), disinfectants (benzalkonium chloride and chlorhexidine) (3.2 µg/ml, 6.4 µg/ml, 12.8 µg/ml, 25.6 µg/ml, 51.2 µg/ml) respectively. The results are expressed as the ratio of the number of colony forming units obtained from LB cultures containing different concentrations of NaCl, bile and disinfectants to the number of colony forming units obtained from control cultures (LB agar alone). These experiments were performed at least three times.

### Heat Shock Challenge Assay

The wild type and mutant was exposed to different temperatures for heat shock assay such as 30°C, 60°C and 72°C, survival was checked on LB and LB Kan (100 µg/ml) plates respectively.

### Oxidative and Nitrostative Stress Tolerance Assay

In this susceptibility test, small Whatman 3 MM paper disks (6 mm) were impregnated with different amount of H_2_O_2_ (10 µl of 3%, 10% and 30%) and later air dried as reported before [50]. The different strains were grown to the mid-log phase (OD_600 nm_ 0.2) and were uniformly spread over an LB agar plate. Next, filter paper disks impregnated with specific concentrations of H_2_O_2_ was placed at the centre on to the agar surface. The culture was then incubated at 37°C for 12–24 hours. The diameter of a zone of inhibition was measured (in millimeters) which is a qualitative measure of the inhibitory activity of a compound. The data represents the distances from the edge of the disks to the end of the clear zone, where growth begins. Each experiment was repeated at least three times. The sensitivity of cells to oxidative stress was tested by exposing stationary-phase bacteria diluted in LB medium (OD_600 nm_ 0.2) at 37°C to 0.07894 mM, 0.7894 mM, 1.5788 mM, 2.3682 mM and 3.1576 mM for 1 h. Viable cells were counted by plating them onto agar plates before and after exposure to H_2_O_2_, and results are expressed as survival percentages.

Sodium nitroprusside (SNP) and acidified nitrite were used to generate nitrostative stress to check cell growth against these NO releasing agents [24]. Growth of cultures against SNP was determined as described previously [21]. Briefly, different strains were grown aerobically in LB medium up to OD_600 nm_ of 0.2. The cells were then treated with different concentrations of SNP and growth was monitored at OD_600 nm_ at an interval of every one hour. To check the response of cultures against acidified nitrite, growth profile of different strains, were determined at pH 6.0 and 7.0 in buffered LB medium supplemented with 10 and 30 mM sodium nitrite and compared with the WT by observing OD_600 nm_ periodically.

### Kirby Bauer Disc Diffusion Assay

Strains in this study were examined for resistance to different antibiotics by using commercial discs (Hi Media, Bombay, India) as described previously [21], according to the interpretation criteria recommended by Clinical and Laboratory Standards Institute CLSI [51].

### Determination of MIC

MIC of antibiotics was tested using E-strips. Interpretation was done as per the criteria approved by CLSI. *E. coli* ATCC 25922 was used as a reference strain (control) as recommended.

### OMP Preparation

OMPs were purified by the method as described previously [13]. Cells were harvested by centrifugation 5000 g for 15–20 mins and were suspended in 50 mM Tris-HCl buffer (pH 7.4) containing 5 mM phenylmethylsulfonyl fluoride and sonicated for 15 mins. The crushed material was treated with DNase and RNase (each at 100 µg/ml), and the unbroken cells were removed by centrifugation (10,000 × g for 10 min). The crude envelope fraction was collected from the supernatant by centrifugation at 105,000× g for 1 h at 4°C. The pellet containing the crude envelope fraction was treated with 0.5% (wt/vol) sarkosyl (Sigma) solution to selectively solubilise the inner membrane part. The insoluble OM fraction was recovered as pellet by centrifugation at 105,000× g for 1 h at 4°C. The pellet was washed and stored at −20°C until used. Protein contents of membrane preparations were determined by the method of bicinchoninic acid (BCA) method (Pierce BCA protein assay kit, cat# 23225) with bovine serum albumin (BSA) (Sigma) as standard.

### Gene Cloning, Expression, Purification and Electrophoretic Mobility Shift Assays (EMSA)

The DNA-binding transcriptional regulator gene *phoB* was amplified using gene specific primers, *phoB-F* and *phoB-R* has NdeI and BamHI sites of the pET28C vector to generate an N-terminal His_6_-PhoB fusion protein. All clones were confirmed by sequencing and transformed into *E. coli* BL21 (DE3). After induction with 0.2 mM isopropyl 1-thio-β-d-galactopyranoside, PhoB protein was purified through Qiagen Ni^2+^nitrilotriacetic acid columns. The protein was dialysed using Tris buffer pH 8.0. The ability of PhoB to bind *kpnO* promoter was tested by EMSA. The *kpnO* promoter region was amplified using prom*kpnO*-F and prom*kpnO*-R primers (Table 3) and subjected to EMSA with purified PhoB protein. Briefly, end-labelled (using [γ-^32^P] ATP) PCR products were incubated with increasing concentrations (in a range of 50 nM to 500 nM) of PhoB in binding buffer (10 mM Tris-HCl, pH 8.0, 2 mM EDTA, 0.5 mM DTT, 50 mM NaCl, 10% glycerol, and 1 µg of poly(dI·dC). The complexes were run on 5% native polyacrylamide gel electrophoresis (PAGE) gels for 2 h. The gel was then dried and exposed to the phosphor screen for image analysis. To confirm that the interaction between PhoB and the promoter region of *kpnO* was specific, experiments with competitive (specific: 10 fold excess of cold promoter and non-specific: poly dI-dC) and non-competitive inhibitor (BSA) were also performed.

### RNA Isolation and Real-time Reverse Transcription PCR (RT-PCR)

Total RNA was extracted from the log-phase cultures of wild-type and *phoB* ^KP^ mutant using the RNeasy Mini Kit (Qiagen) according to the manufacturer's instructions. Total RNA was digested with DNase I to ensure the removal of contaminating genomic DNA prior to cDNA synthesis. Aliquots of 500 ng of DNase I treated total RNA served as template for complementary DNA (cDNA) synthesis using SuperScript III Reverse Transcriptase (Invitrogen). The cDNA samples were diluted 1∶100 and 2 µL was used per 25 µL quantitative PCR reaction for *kpnO* and were performed using gene specific primers (Table 3). Gene expression levels were monitored by real time RT-PCR using Maxima SYBR Green qPCR master mix (Fermentas) in an iCycler thermal cycler (Bio-Rad) and the melting curve analysis were carried out to confirm amplification of a single product. Total RNA was isolated from at least two separately grown replicate cultures. All real time RT-PCR experiments were performed in triplicate, with *rpoB as* an internal control.

### Caenorhabditis Elegans Killing Assay

Bacterial virulence (both agar and liquid killing) assays were performed using nematode model, *C. elegans* strain Bristol N2 as described previously with slight modifications [52], [53]. To examine the ability of wild-type, mutant and *E. coli* OP50 strains to kill *C. elegans*, bacterial lawns of the *K. pneumoniae* and an *E. coli* control strain were prepared on nematode growth (NG) media and incubated at 37°C for 6 h. The plates were kept at room temperature for 1 hr and then seeded with L4-stage worms (25 to 30 per plate). Further the seeded plates were incubated at 25°C and examined for live worms under a stereomicroscope (Leica MS5) after every 24 hours. When the worm did not react to touch it was considered dead. At least five replicates repeated three times were performed for each selected strain.

### Bioinformatic Analysis and Statistical Analysis

Multiple sequence alignments were carried out using the Clustal program www.ebi.ac.uk Homology searches, similarities and identities analysis and conserved domain architecture analysis were performed using NCBI Internet server [54], Simple Modular Architecture Research Tool (SMART) www.smart.embl-heidelberg.de and NCBI conserved domain search. All data are presented as means ± the standard error of the mean. Plotting and calculation of the standard deviation was performed in Microsoft Excel. Statistical analysis was performed on crude data by using a paired Student t test. P values of <0.05 were considered significant.

## Supporting Information

Figure S1 **Oxidative stress assays.** Survival of *K. pneumoniae* and Δ*kpnO* strains upon exposure to oxidative stress with 0.07894 mM, 0.7894 mM, 1.5788 mM, 2.3682 mM and 3.1576 mM. After 1 h of treatment with 0.07894 mM hydrogen peroxide, only 47% of Δ*kpnO* cells survived in comparison to 95% of the wild-type cells. The differences between the mutant and its parental wild-type strain are statistically significant (P<0.05) for all H_2_O_2_ concentrations. The standard errors of the means from three independent assays are shown.
(TIF)

Figure S2 **Nitrostative challenge assays.** Growth pattern of WT, Δ*kpnO* in the presence of sodium nitrite. In the presence of 30 mM NO donor, growth kinetics of Δ*kpnO* cells was ∼7.0 fold lower as compared to WT at pH 7.0.
(TIF)

Figure S3 **Growth inactivation assays.** Inactivation assays using ciprofloxacin (0.005 µg/ml). The efflux pump inhibitors CCCP was used at a concentration of 10 µg/ml in the experiment. The mean values of three independent experiments have been used for plotting the graph.
(TIF)

Figure S4 **Nitrostative challenge assays.** Effect of SNP (20 mM and 30 mM) on growth kinetics of WT and Δ*phoB* ^KP^.
(TIF)

Figure S5 **In vitro inactivation assays.** Growth inactivation assays using ciprofloxacin (0.005 µg/ml). The efflux pump inhibitors CCCP was used at a concentration of 10 µg/ml in the experiment. The mean values of three independent experiments have been used for plotting the graph.
(TIF)
