# Supplementary figures and images for: Functional Characterization of a Novel Outer Membrane Porin KpnO, Regulated by PhoBR Two-Component System in Klebsiella pneumoniae NTUH-K2044
Source: PLoS One. 2012 Jul 25;7(7):e41505. doi: 10.1371/journal.pone.0041505 (PMC3405095; doi:10.1371/journal.pone.0041505)

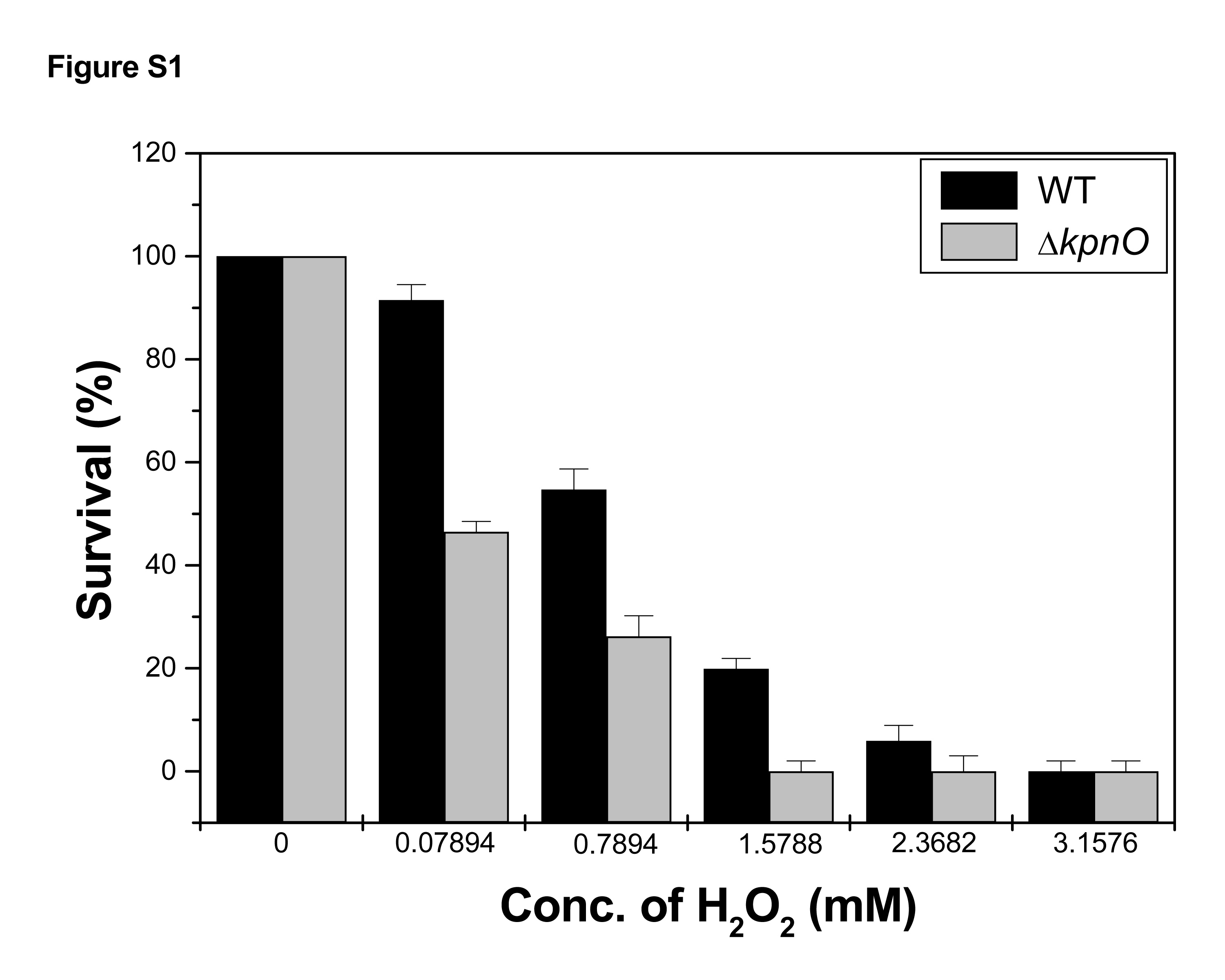

Supplement: Figure S1 — Oxidative stress assays. Survival of K. pneumoniae and ΔkpnO strains upon exposure to oxidative stress with 0.07894 mM, 0.7894 mM, 1.5788 mM, 2.3682 mM and 3.1576 mM. After 1 h of treatment with 0.07894 mM hydrogen peroxide, only 47% of ΔkpnO cells survived in comparison to 95% of the wild-type cells. The differences between the mutant and its parental wild-type strain are statistically significant (P<0.05) for all H2O2 concentrations. The standard errors of the means from three independent assays are shown. (TIF) [file pone.0041505.s001.tif]

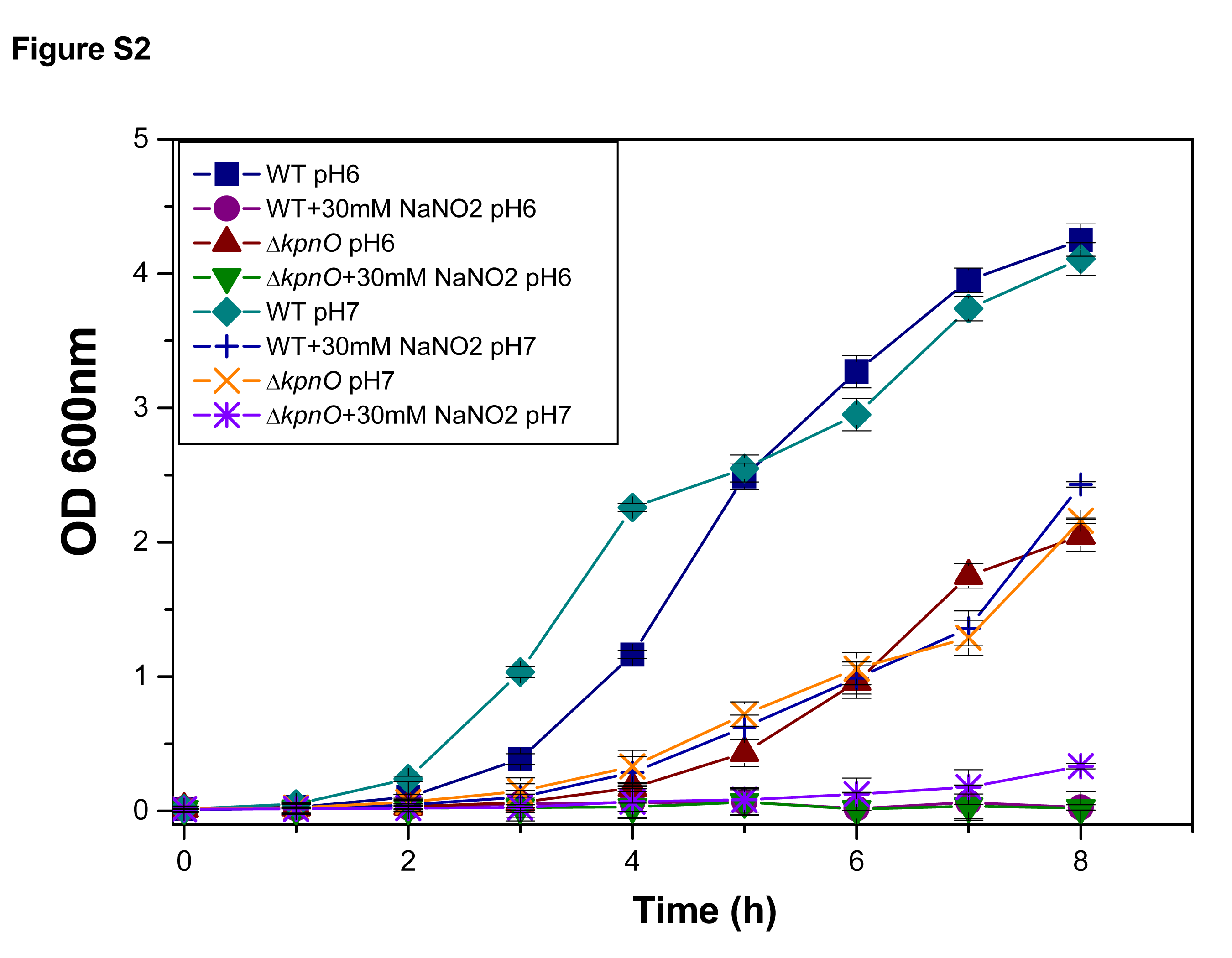

Supplement: Figure S2 — Nitrostative challenge assays. Growth pattern of WT, ΔkpnO in the presence of sodium nitrite. In the presence of 30 mM NO donor, growth kinetics of ΔkpnO cells was ∼7.0 fold lower as compared to WT at pH 7.0. (TIF) [file pone.0041505.s002.tif]

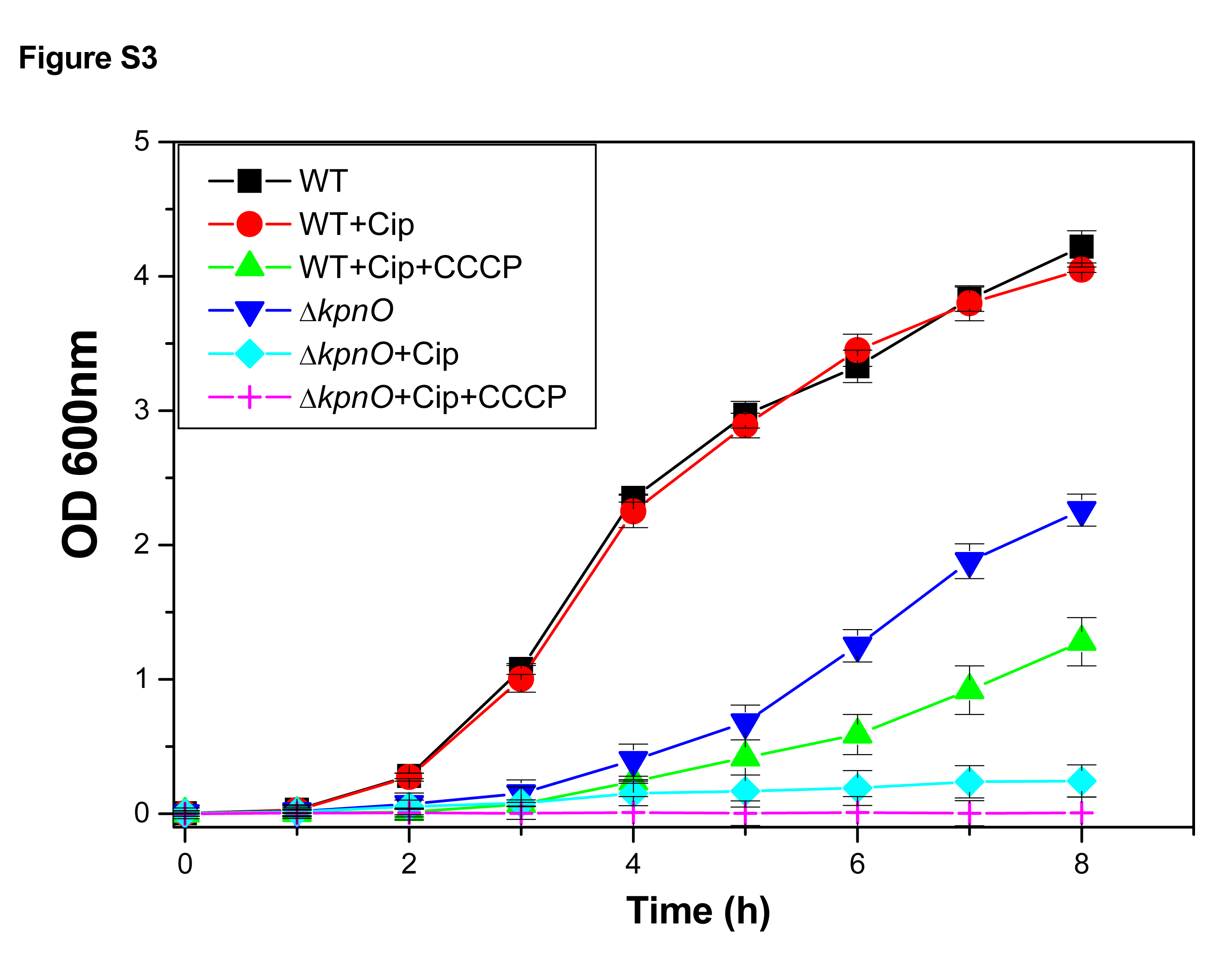

Supplement: Figure S3 — Growth inactivation assays. Inactivation assays using ciprofloxacin (0.005 µg/ml). The efflux pump inhibitors CCCP was used at a concentration of 10 µg/ml in the experiment. The mean values of three independent experiments have been used for plotting the graph. (TIF) [file pone.0041505.s003.tif]

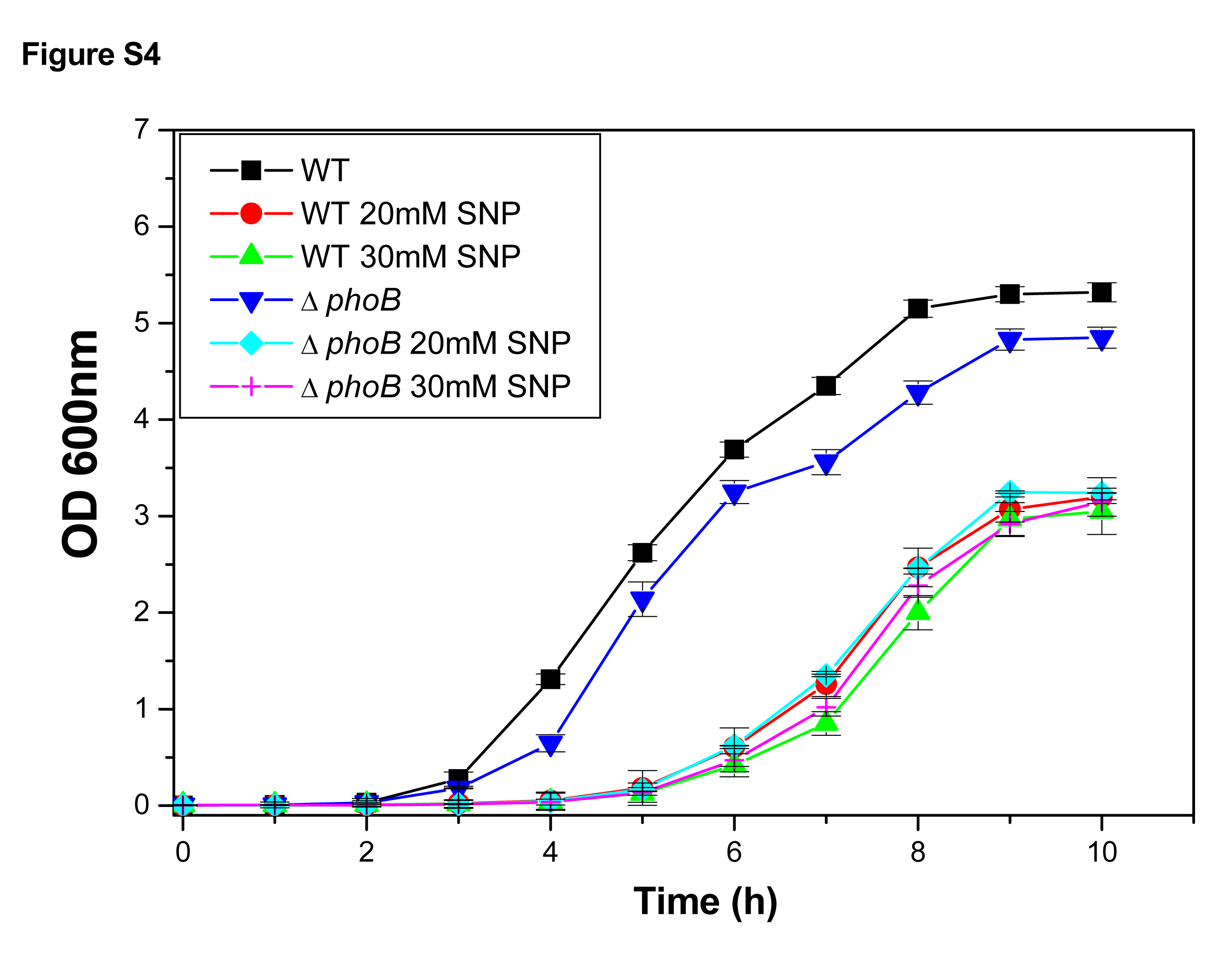

Supplement: Figure S4 — Nitrostative challenge assays. Effect of SNP (20 mM and 30 mM) on growth kinetics of WT and ΔphoB KP. (TIF) [file pone.0041505.s004.tif]

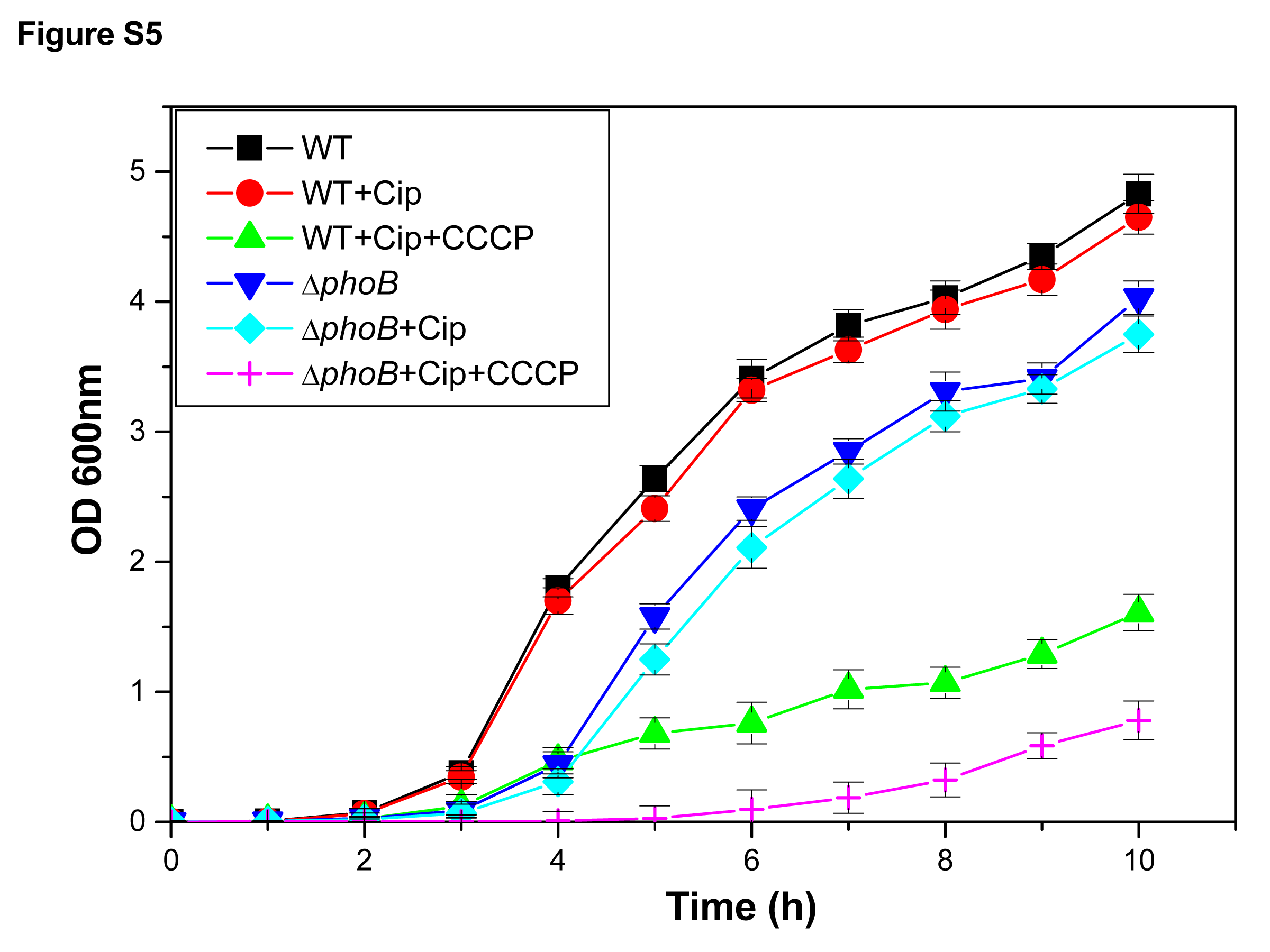

Supplement: Figure S5 — In vitro inactivation assays. Growth inactivation assays using ciprofloxacin (0.005 µg/ml). The efflux pump inhibitors CCCP was used at a concentration of 10 µg/ml in the experiment. The mean values of three independent experiments have been used for plotting the graph. (TIF) [file pone.0041505.s005.tif]
